# Hmgcs2-mediated ketogenesis modulates high-fat diet-induced hepatosteatosis

**DOI:** 10.1016/j.molmet.2022.101494

**Published:** 2022-04-12

**Authors:** Shaza Asif, Ri Youn Kim, Thet Fatica, Jordan Sim, Xiaoling Zhao, Yena Oh, Alix Denoncourt, Angela C. Cheung, Michael Downey, Erin E. Mulvihill, Kyoung-Han Kim

**Affiliations:** 1 University of Ottawa Heart Institute, Ottawa, ON, K1Y 4W7, Canada; 2 Department of Cellular and Molecular Medicine, Faculty of Medicine, University of Ottawa, Ottawa, ON, K1H 8M5, Canada; 3 Department of Pathology and Laboratory Medicine, The Ottawa Hospital, Ottawa, ON, K1H 8M5, Canada; 4 Ottawa Institute of Systems Biology, Ottawa, ON, K1H 8M5, Canada; 5 Gastroenterology and Hepatology, Department of Medicine, The Ottawa Hospital, Ottawa, ON, K1H 8M5, Canada; 6 The Ottawa Hospital Research Institute, Chronic Disease Program, Ottawa, ON, K1Y 4E9, Canada; 7 Department of Biochemistry, Microbiology and Immunology, Faculty of Medicine, University of Ottawa, Ottawa, ON, K1H 8M5, Canada

**Keywords:** Ketogenesis, Hmgcs2, NAFLD, Lipid accumulation, AcAc, acetoacetate, ANOVA, analysis of variance, ASO, antisense oligonucleotide, AUC, area under the curve, β-OHB, β-hydroxybutyrate, FAO, fatty acid oxidation, GLP1R, glucagon-like peptide-1 receptor, GTT, glucose tolerance test, H&E, hematoxylin & eosin, HET, heterozygous, HFD, high-fat diet, HMG-CoA, β-hydroxy β-methylglutaryl-CoA, HMGCS2, 3-hydroxymethylglutaryl-CoA synthase 2, HMGCL, HMG-CoA lyase, HOMA-IR, homeostasis model assessment-estimated insulin resistance, IHC, immunohistochemistry, ITT, insulin tolerance test, KO, knockout, mHL, mitochondrial HMG-CoA lyase deficiency, mHS, mitochondrial HMG-CoA synthase deficiency, NAFLD, non-alcoholic fatty liver disease, NAS, NAFLD activity score, NASH, non-alcoholic steatohepatitis, OE, overexpression, Plin2, perilipin 2, PSR, picrosirius red, SGLT2, sodium-glucose transport protein 2, EM, standard error of the mean, T2D, type 2 diabetes, TCA, tricarboxylic acid, WT, wild-type

## Abstract

**Objective:**

Aberrant ketogenesis is correlated with the degree of steatosis in non-alcoholic fatty liver disease (NAFLD) patients, and an inborn error of ketogenesis (mitochondrial HMG-CoA synthase deficiency) is commonly associated with the development of the fatty liver. Here we aimed to determine the impact of Hmgcs2-mediated ketogenesis and its modulations on the development and treatment of fatty liver disease.

**Methods:**

Loss- and gain-of-ketogenic function models, achieved by *Hmgcs2* knockout and overexpression, respectively, were utilized to investigate the role of ketogenesis in the hepatic lipid accumulation during postnatal development and in a high-fat diet-induced NAFLD mouse model.

**Results:**

Ketogenic function was decreased in NAFLD mice with a reduction in Hmgcs2 expression. Mice lacking *Hmgcs2* developed spontaneous fatty liver phenotype during postnatal development, which was rescued by a shift to a low-fat dietary composition via early weaning. *Hmgcs2* heterozygous adult mice, which exhibited lower ketogenic activity, were more susceptible to diet-induced NAFLD development, whereas *HMGCS2* overexpression in NAFLD mice improved hepatosteatosis and glucose homeostasis.

**Conclusions:**

Our study adds new knowledge to the field of ketone body metabolism and shows that Hmgcs2-mediated ketogenesis modulates hepatic lipid regulation under a fat-enriched nutritional environment. The regulation of hepatic ketogenesis may be a viable therapeutic strategy in the prevention and treatment of hepatosteatosis.

## Highlights

• Ketogenic function was reduced in NAFLD mice with decreased Hmgcs2 expression.
• Postnatal mice lacking *Hmgcs2* showed ketogenic deficiency and spontaneously developed fatty liver disease.
• Early weaning rescued the hepatosteatosis in postnatal *Hmgcs2* knockout mice.
• Insufficient ketogenic activity increased susceptibility to diet-induced fatty liver disease in adult mice.
• *HMGCS2* overexpression improved the high-fat diet-induced fatty liver disease in adult mice.

## 1 Introduction

Non-alcoholic fatty liver disease (NAFLD), characterized by an abnormal and excessive accumulation of intracellular lipids in hepatocytes, is the most common chronic liver disease, with an approximate global prevalence of 25% [1,2]. NAFLD is driven by multiple factors, including lifestyle, genetics (i.e., Patatin-like phospholipase domain-containing protein 3, *PNPLA3*) and other metabolic conditions, such as Type 2 Diabetes (T2D) and obesity. If not managed or treated, a proportion of NAFLD patients can develop the more severe form of fatty liver disease, non-alcoholic steatohepatitis (NASH), which is associated with increased metabolic dysfunction, inflammation, fibrosis, and a greater risk of progression to the irreversible stages of cirrhosis, liver failure, and hepatocellular carcinoma. Current drugs targeting metabolic conditions, such as sodium-glucose transport protein 2 (SGLT2) inhibitors or the glucagon-like peptide-1 receptor (GLP1R) agonists for diabetes, have also shown improvements in NAFLD patients [3,4], yet a lack of liver-specific pharmacological agents limits the effective management and treatment of fatty liver disease.

The accumulation of hepatic fat is associated with an imbalance between lipid deposition and clearance pathways in the liver. Insulin resistance-mediated increases in adipocyte lipolysis and non-esterified fatty acid flux to the liver as well as *de novo* lipogenesis promote hepatic lipid accumulation, whereas impaired fatty acid oxidation (FAO) and reduced very-low-density lipoprotein secretion prevent hepatic lipid clearance [[5], [6], [7]]. In particular, mitochondrial β-oxidation is a major pathway of lipid disposal in the liver. Through this pathway, repeated reactions convert fatty acids into acetyl-CoA, which can then enter the tricarboxylic acid (TCA) cycle for terminal oxidation or be converted into energy-carrying ketone bodies, primarily acetoacetate (AcAc) and β-hydroxybutyrate (β-OHB), through ketogenesis [8,9].

3-hydroxymethylglutaryl-CoA synthase 2 (HMGCS2) is the key rate-limiting enzyme of ketogenesis; it catalyzes the breakdown of β-oxidation-derived acetyl-CoA and acetoacetyl-CoA to produce β-hydroxy β-methylglutaryl-CoA (HMG-CoA) and free Coenzyme A (CoA). HMG-CoA is cleaved by HMG-CoA lyase (HMGCL) to generate AcAc, which is further reduced to β-OHB through the enzymatic activity of D-3-hydroxybutyrate dehydrogenase 1 (BDH1). The ketone bodies, AcAc and β-OHB, are transported to extrahepatic tissues (i.e., brain, skeletal muscle) where they are terminally oxidized for fuel [9]. These ketone bodies serve as critical energy substrates during settings of carbohydrate deprivation, such as fasting and exercise. Under these ketotic conditions, blood ketone levels that are normally within the limit of 0.1 mmol/L, can reach up to 7–8 mmol/L [10]. In addition, a fat-enriched diet can increase ketogenesis. For example, high-fat diet (HFD) feeding for 16 weeks in mice elevates ketone body synthesis [11,12]. Moreover, the shift from a prenatal carbohydrate-enriched environment (dependent on placental glucose transfer) to a postnatal fat-enriched environment (dependent on breast milk), promotes ketogenesis and a consequential reliance on ketone bodies as primary energy substrates in neonates [[13], [14], [15], [16], [17]]. In addition to their fat-metabolizing and energy-generating capacity, ketone bodies provide other health benefits, including protection from inflammation and oxidative stress [9,18,19]. As such, ketogenic diets [[20], [21], [22], [23]] and ketone ester formulations [24] have gained popularity for stimulating weight loss [10,25] and improving metabolic disorders (i.e., T2D, obesity, NAFLD).

Recent studies have shown an association between impaired ketogenesis and fatty liver disease. In particular, dysregulated ketogenesis is correlated with the degree of hepatic lipid accumulation in NAFLD patients [[26], [27], [28]], and infants with an inborn error of ketogenesis (mitochondrial HMG-CoA synthase deficiency) commonly develop fatty liver [[29], [30], [31], [32]]. In addition, ketogenic insufficiency through antisense oligonucleotide (ASO)-mediated *Hmgcs2* knockdown in adult mice results in hepatic inflammation and injury, characteristics of advanced NAFLD (e.g., NASH) [33,34]. However, it remains elusive as to what is the causative genetic factor underlying impaired ketogenesis in NAFLD, what is the necessity in the relationship between ketogenesis and a fat-enriched diet during fatty liver development, and whether the degree of ketogenic activity is associated with susceptibility to NAFLD development and progression. Importantly, it is not yet known whether increased ketogenesis is sufficient to reverse NAFLD.

To address these questions, in the present study, we employ *Hmgcs2* loss- and gain-of-function mouse models to investigate a causal relationship of deficient and active ketogenesis in NAFLD development and treatment, respectively. Notably, a change in dietary composition rescued the fatty liver phenotype of postnatal *Hmgcs2* knockout mice, whereas increased ketogenic function through *HMGCS2* overexpression improved hepatosteatosis and associated metabolic dysfunction in NAFLD mice. Together, our study adds new knowledge to the field of ketone body metabolism and suggests a viable therapeutic strategy involving ketogenesis activation in preventing and treating NAFLD.

## 2 Materials & methods

### 2.1 Animal maintenance, diets, and treatment

All animal experiments were performed in accordance with protocols (#2950 and #2962) approved by the Animal Care Committee in the Animal Care and Veterinary Service (ACVS) at the University of Ottawa and conformed to the standards of the Canadian Council on Animal Care. All mice were housed in standard vented cages in temperature- and humidity-controlled rooms with 12-hour light–dark cycles (21–22 °C, 30–60% humidity for housing), and free access to water. Mice were provided with either standard chow (SC) in which 22% of calories were from fat, 55% from carbohydrates and 23% from protein (2019 Teklad Global Diet; Envigo) or high-fat diet (HFD) in which 45% of calories were from fat, 35% from carbohydrates and 20% from protein (D12451; Research Diets).

The early weaning protocol was adapted from previous studies [35,36]. The day of birth was termed as postnatal day 0 (p0). Post-birth and during the suckling period, the mother and pups were undisturbed, except briefly on a weekly basis to change cages. At p14, pups were prematurely weaned and provided with a standard chow in the form of soaked cubes placed on the cage floor for easy access. Mice were maintained up until 1-week post-weaning or p21. Control suckling mice were kept with the mother until p21.

### 2.2 Generation of *Hmgcs2* knockout mouse

Mice lacking the *Hmgcs2* gene, C57BL/6N-*Hmgcs2*^*em1(IMPC)Tcp*^ (*Hmgcs2* knockout; *Hmgcs2*-KO) were generated at The Centre for Phenogenomics (TCP; Toronto, Canada) as previously described [37]. Briefly, C57BL/6NCrl zygotes were electroporated with Cas9 ribonucleoprotein complexes with single guide RNAs having spacer sequences of CTAATATTACGCTTGAAAGT targeting the 5′ side and GTGCCTGACTGTAGATGAGA targeting the 3′ side of a critical region (Supplementary Fig. 1A). This resulted in a 1000-bp deletion, Chr3:98290599 to 98291598_insT (GRCm38). All procedures involving animals were performed in compliance with the Animals for Research Act of Ontario and the Guidelines of the Canadian Council on Animal Care. Founders were identified by endpoint PCR using PCR primers flanking the deletion target, Fwd: 5′-AATTCAGAATTCAAAGCTACCTGGG-3′ and Rev: 5′-CTCAGAGGCTCCAGGAGATTAAT-3′. The wild-type allele would produce a 2,208 base-pair (bp) amplicon and the deletion fragment a 1,209 bp amplicon. Founders were backcrossed to wild-type C57BL/6NCrl mice, and N1 progeny was identified using the same PCR. Sanger sequencing of deletion amplicons from N1 mice was used to identify the definitive deletion sequence. Mice were maintained by crossing *Hmgcs2*^*em1(IMPC)Tcp*^ heterozygotes with C57BL/6NCrl wild-type mice. This strain is available from the Canadian Mouse Mutant Repository at TCP.

Genotyping of *Hmgcs2*-KO mice was performed using Dream Taq™ Hot Start Green PCR Master Mix (Thermo Scientific™) with Common forward primer (FP1), CCTCCTAGACATGCATGCCC; Wild-type (WT) reverse primer (RP1), ACCCAACCAATAATGTGGCA; and Knockout (KO) reverse primer (RP2), AGTTGCTCTTGCCAGTGGTT. Band sizes for WT and KO were 327 and 203 bp, respectively (Supplementary Fig. 1B).

### 2.3 Adenovirus-mediated *HMGCS2* overexpression in mice

The *HMGCS2* overexpression (OE) mouse model was achieved by a single intravenous injection (1.0 × 10^9^ PFU) of adenovirus vectors encoding human *HMGCS2* (Ad-*HMGCS2*, ADV-211285; Vector BioLabs) through the tail vein. Ad-*GFP* (#1060; Vector BioLabs) was used for the control group.

**Figure 5:**
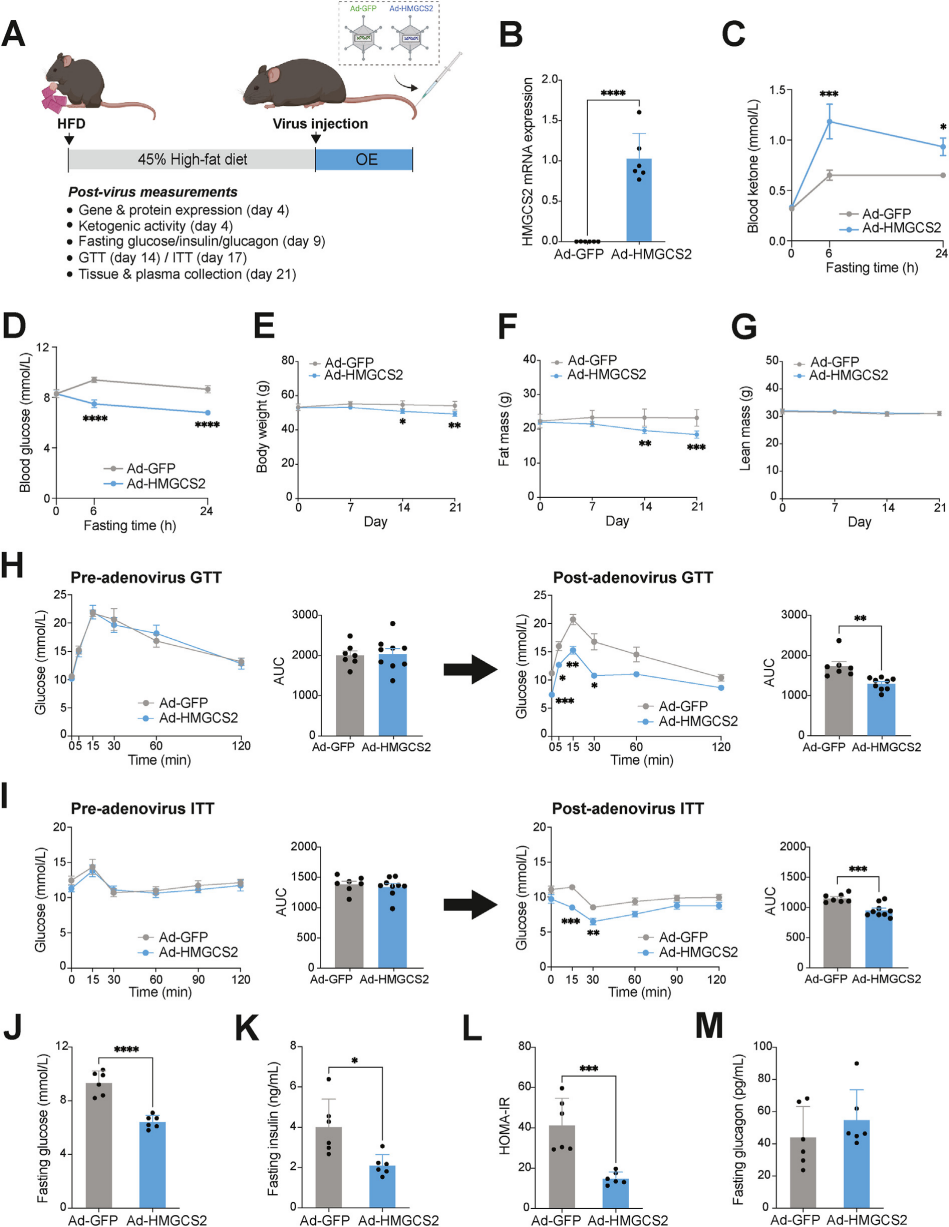
**Ketogenesis activation through *HMGCS2* overexpression confers metabolic improvements in HFD-induced NAFLD mice.** (A) Schematic illustration of the *HMGCS2* overexpression experiment, using HFD-induced NAFLD mice that were intravenously administered with adenovirus expressing *GFP* (Ad-*GFP*) or *HMGCS2* (Ad-*HMGCS2*). (B) Human *HMGCS2* mRNA expression in the Ad-*HMGCS2* mouse livers, compared to Ad-*GFP* controls. (C) Blood ketone and (D) glucose levels at baseline and 6- and 24-hour post-fasting, measured at day 4 post-virus injection (n = 6/group). Weekly measurements of (E) body weight, (F) fat mass and (G) lean mass of mice for 3 weeks post-virus administration. Pre- and post-virus (H) glucose tolerance test (GTT) and (I) insulin tolerance test (ITT) and their respective area-under the curve (AUC) (Ad-*GFP*, n = 7; Ad-*HMGCS2*, n = 9). (J) Blood glucose, (K) plasma insulin, (L) Homeostatic Model Assessment for Insulin Resistance (HOMA-IR), and (M) plasma glucagon of Ad-*GFP* and Ad-*HMGCS2* mice, measured at 6-hour fasting (n = 6/group). Data are represented as mean ± SEM. Statistical analysis was performed by student's *t*-test or two-way repeated measures ANOVA. ∗*P* ≤ 0.05; ∗∗*P* ≤ 0.01; ∗∗∗*P* ≤ 0.001; ∗∗∗∗*P* ≤ 0.0001. (Created with BioRender.com).

Four independent rounds of *HMGCS2*-OE experiments (EXP) were performed in this study. EXP #1: adenovirus injection in HFD-fed (24 weeks) C57BL/6J mice for 2 weeks (Ad-*GFP* = 3; Ad-*HMGCS2* = 4). This experiment was performed as preliminary assessments of *HMGCS2* overexpression and metabolic phenotypes; EXP #2: adenovirus injection in HFD-fed (32 weeks) C57BL/6J mice for 4 days (Ad-*GFP* = 6; Ad-*HMGCS2* = 6). This experiment was performed to examine HMGCS2 protein expression; EXP #3: adenovirus injection in HFD-fed (45 weeks) C57BL/6J mice for 9 days (Ad-*GFP* = 6; Ad-*HMGCS2* = 6). This experiment was performed to examine ketogenic activities and glucose metabolism in mice; EXP #4: adenovirus injection in HFD-fed (34 weeks) C57BL/6J mice for 3 weeks (Ad-*GFP* = 7; Ad-*HMGCS2* = 9). This experiment was conducted for the complete assessment of metabolic phenotypes and hepatosteatosis. Human *HMGCS2* gene expression in relation to endogenous mouse *Hmgcs2* was measured in various metabolic tissues collected from EXP #1 (Supplementary Figs. 5A and B) and EXP #2 (Figure 5B). Hmgcs2 protein expression was examined in livers collected from EXP #2 (Supplementary Fig. 5C). Ketogenic activity and blood glucose levels upon fasting were assessed on day 4 post-virus injection in EXP #3, prior to the development of visible metabolic phenotypes (e.g., body weight changes) (Figure 5C–D). Fasting glucose, insulin and glucagon were measured at day 9 post-virus injection in EXP #3 (Figure 5J–M). Glucose and insulin tolerance tests (GTT and ITT) were performed on days 14 and 19 post-virus injection, respectively, in EXP #4 (Figure 5H–I). Tissue and plasma collections for NAFLD assessments were performed at day 21 post-virus injection in EXP #4 (Figure 5E–G; Figure 6A–I; Supplementary Figs. 6A–D).

### 2.4 Mouse metabolic phenotyping

Body fat and lean masses of mice were measured using the EchoMRI-3-in-1 machine (Echo Medical Systems, Houston, TX, USA), as previously described [38]. Blood glucose and ketone levels were measured using a glucometer (Contour® Next One; Ascensia Diabetes Care Inc.) and ketone meter (Freestyle Optium Neo and β-ketone Test Strips; Abbott Diabetes Care Ltd.), respectively. Glucose and insulin tolerance tests (GTT and ITT) were performed by intraperitoneal injection of glucose (1 mg/g of body weight) and insulin (0.65 mU/g of body weight, Humalog®; Eli Lilly Canada Inc) after an overnight (15-hour) and 6-hour fasting with *ad libitum* access to water, respectively [39]. Glucose levels were measured using blood samples from the tail vein at the indicated intervals. Plasma insulin (80-INSMSU-E01; ALPCO) and glucagon (#81518, Crystal Chem) were quantified using ELISA kits. The homeostasis model assessment-estimated insulin resistance (HOMA-IR) was calculated according to the formula: fasting glucose (mmol/L) × plasma insulin (μU/mL)/22.5. Plasma alanine aminotransferase (ALT) was measured using ALT Activity Assay Kit (MAK052; Sigma–Aldrich). For liver triglyceride measurement, frozen liver tissues were homogenized in 500 μl of 5% NP-40/ddH_2_O using a Mini-Beadbeater (BioSpec Products) and solubilized samples were quantified using a colorimetric Triglyceride Assay Kit (Ab65336; Abcam). Liver collagen was quantified using the Total Collagen Assay Kit (Ab222942; Abcam) from samples obtained via liver tissue homogenization and hydrolysis.

### 2.5 Histological analysis of liver tissue

Liver tissue samples were fixed in 4% paraformaldehyde, embedded in paraffin, and cut to 5 μm thick sections. Lipid droplets were visualized by staining with hematoxylin and eosin (H&E) or immunohistochemistry with anti-Perilipin 2 (Plin2) antibody (1:150, NB110-40877; Novus Biologicals) and the VECTASTAIN Elite ABC reagent (PK-6100; Vector Laboratories). Collagen deposition was visualized by picrosirius red (PSR) staining. Slide images were captured using the Aperio VERSA 8 Scanner (Leica Biosystems).

Stained liver sections were graded and staged by a certified histopathologist for histological features according to the NASH Clinical Research Network Grading and Staging System [40]. A NAFLD activity score (NAS) was obtained from H&E-stained liver sections by summing the individual semi-quantitative scores of steatosis (score 0–3), lobular inflammation (score 0–3), and ballooning (score 0–2). Liver fibrosis was scored separately from PSR-stained sections (stage 1–4), according to the Batts and Ludwig scoring system [41].

### 2.6 Cell culture

HepG2 hepatoma cells were cultured in EMEM supplemented with lipoprotein-free fetal bovine serum (FBS) at 37 °C with 5% CO_2_ and were allowed to reach 70–80% confluency. Cells were then treated with 1.5 mM water-soluble oleic acid (#01257; Sigma) for 24 hours, followed by adenovirus infection (7.5 MOI) of Ad-*GFP* and Ad-*HMGCS2*. After 24 hours, cells were harvested for downstream molecular and imaging analysis. To assess lipid accumulation, cells were fixed in 10% formalin and stained in freshly prepared Oil Red O solution (0.5% in isopropanol, Sigma). Stained cells were imaged using a light microscope. For lipid quantification, the dye was extracted from the stained cells by isopropanol with 4% NP-40, and absorbance was determined at 490 nm.

### 2.7 RNA isolation and quantitative PCR

Total RNA was extracted using TRIzol™ Reagent (Invitrogen) and further purified using the PureLink™ RNA Mini Kit (Invitrogen). Tissue samples were homogenized using a Mini-Beadbeater (BioSpec Products). Complementary DNA (cDNA) was synthesized from 1 or 2 μg of RNA using the High-Capacity cDNA Reverse Transcription Kit with RNaseOUT™ (Invitrogen). A final concentration of 5 ng/μL cDNA was used for quantitative PCR (qPCR). Gene expression assay was conducted using Power SYBR Green Master Mix (Thermofisher) on Quant studio 5 (Applied Biosystems), and relative cycle threshold (CT) values were normalized by housekeeping TATA-Box Binding Protein (*Tbp*) gene. For gene-tissue expression analyses, all samples were run on a single qPCR plate for direct comparison of the gene expression level. All primer sequences are indicated in Supplementary Table 1**.**

### 2.8 Western blot

Tissue or cell lysates were prepared using RIPA Lysis and Extraction Buffer (#89900; Thermo Scientific™) and protease inhibitor (#78442; Thermo Scientific™). After protein quantification using the Pierce Rapid Gold BCA Protein Assay Kit (#A53225; Thermo Scientific™), 10 μg of *in vivo* protein samples or 75 μg of *in vitro* protein samples were used for gel electrophoresis (10% TGX Stain-Free FastCast Acrylamide, Bio-Rad) and blotted with anti-Hmgcs2 antibody (1:500–1:1000, sc-393256; Santa Cruz Biotechnology) and β-tubulin antibody (1:500, ab6046; Abcam). Hmgcs2 expression was normalized to total protein loaded on the gel, assessed by stain-free total protein measurement (Bio-Rad). Blots were developed using Clarity™ Western ECL Substrate (#170–5060; Bio-Rad) and Bio-Rad Imager. Densitometry measurement was completed by Image Lab 6.1 Software (Bio-Rad).

### 2.9 Statistical analysis

All data are presented as mean ± standard error of the mean (SEM). Statistical differences between the means of two or more groups were tested using unpaired two-tailed Student's *t-*test or one-way, two-way or two-way repeated-measures analysis of variance (ANOVA). *P* values less than 0.05 were considered statistically significant. Mendelian inheritance was statistically determined using the Chi-square test comparing the total observed mouse numbers of each *Hmgcs2* genotype to expected Mendelian frequencies. The survival curve of postnatal *Hmgcs2* mutant mice was analyzed using the Kaplan–Meier method and statistically compared with the log-rank test. All statistical analyses were performed with Prism 9.0 software (GraphPad Software).

## 3 Results

### 3.1 HFD-induced NAFLD mice present with impaired ketogenesis and Hmgcs2 expression

**Figure 1:**
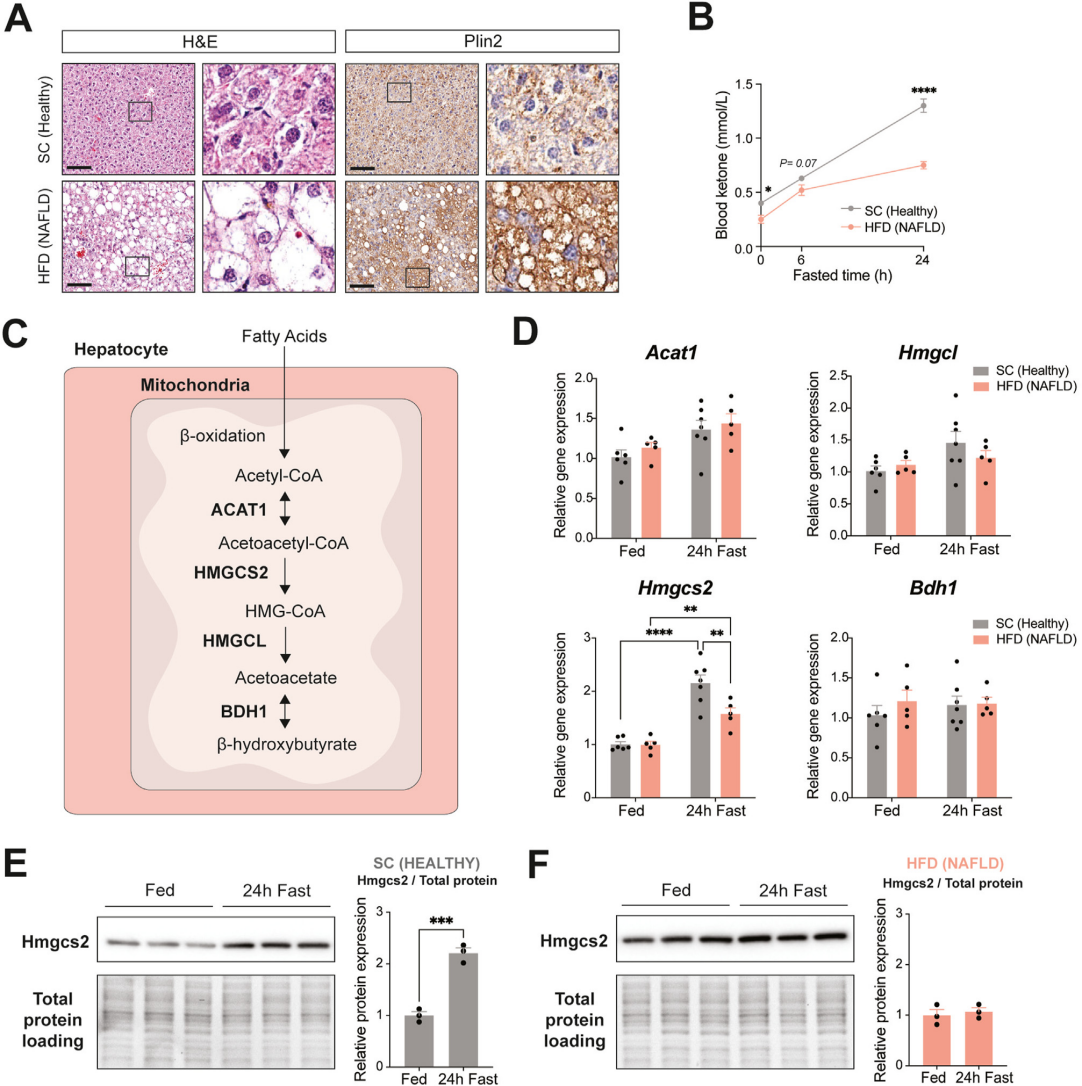
**NAFLD mice exhibit impaired fasting-induced ketogenesis due to abnormal Hmgcs2 expression in the liver.** (A) Hematoxylin & Eosin (H&E) and anti-perilipin 2 (Plin2) immunohistochemical (IHC) staining of liver sections from healthy mice fed standard chow (SC) (22% kcal fat, 55% kcal carbohydrates, 23% kcal protein) and NAFLD mice fed high-fat diet (HFD) (45% kcal fat, 35% kcal carbohydrates, 20% kcal protein) for 32 weeks. Scale bar = 100 μm. Boxes indicate regions of higher magnification. (B) Blood ketone body levels at baseline and 6- and 24-hour post-fasting (SC, n = 7; HFD, n = 10). (C) Schematic of the hepatic ketogenic pathway with key metabolites and enzymes. (D) mRNA expression analysis of ketogenic enzymes in the liver, including *Acat1*, *Hmgcl*, *Hmgcs2*, and *Bdh1*, in fed (SC, n = 6; HFD, n = 5) and 24-hour fast (SC, n = 7; HFD, n = 5). Hmgcs2 protein expression and quantification in (E) SC-fed healthy and (F) HFD-fed NAFLD mouse livers in fed and 24-hour fast (n = 3/group). Data are represented as mean ± SEM. Statistical analysis was performed by student's *t*-test and two-way or two-way repeated measures ANOVA. ∗*P* ≤ 0.05; ∗∗*P* ≤ 0.01; ∗∗∗*P* ≤ 0.001 ∗∗∗∗*P* ≤ 0.0001.

In addition to the altered metabolic pathways in NAFLD pathogenesis (i.e., lipid uptake, secretion, oxidation, synthesis) [42,43], recent studies have demonstrated that NAFLD patients exhibit dysregulated ketogenesis, which is correlated with the severity of the disease (i.e., hepatic steatosis) [26]. To study NAFLD-related impaired ketogenesis in a preclinical model, we established NAFLD in mice by 32 weeks of high-fat diet (HFD; 45% kcal fat) feeding and compared them with healthy mice fed laboratory standard chow (SC; 22% kcal fat). H&E staining and Perilipin 2 (Plin2) immunostaining of liver sections confirmed HFD-induced severe hepatosteatosis, as these mice showed hypertrophied hepatocytes with significant accumulation of lipids, particularly microvesicular steatosis, associated with advanced NAFLD and characterized by the build-up of numerous small lipid droplets surrounding centrally located nuclei within hepatocytes [44] (Figure 1A). Importantly, in contrast to SC-fed healthy mice showing fasting-induced ketogenesis with blood ketone bodies (β-OHB) levels up to 1.30 ± 0.06 mmol/L at 24-hour fasting, HFD-fed NAFLD mice failed to increase blood ketone levels beyond 0.75 ± 0.03 mmol/L (Figure 1B). This result suggests a suppressed ketogenic response in NAFLD mice, corroborating recent findings in NAFLD patients [26,27].

Next, to understand the molecular contribution underlying the altered ketogenic response of NAFLD mice, hepatic expression of ketogenic pathway genes, such as *Acat1*, *Hmgcs2*, *Hmgcl* and *Bdh1*, was examined (Figure 1C). Among these, only *Hmgcs2*, known to be the rate-limiting enzyme of ketogenesis [9], was significantly increased following 24-hour fasting in healthy mice (2.1-fold, *P* < 0.0001) and to a comparatively lesser extent (*P* = 0.006) in mice with NAFLD (1.6-fold, *P* = 0.007) (Figure 1D). Furthermore, quantitative analysis of Western blot consistently showed that fasting increased hepatic Hmgcs2 protein expression (2.2-fold, *P* = 0.0006) in healthy mice (Figure 1E), whereas no similar fasting-induced changes were observed in NAFLD mice (Figure 1F). Together, these results suggest that mice with NAFLD have reduced ketogenic function associated with a blunted response of the fate-committing ketogenic enzyme, Hmgcs2.

### 3.2 Genetic deletion of *Hmgcs2* promotes spontaneous fatty liver development in postnatal mice

**Figure 2:**
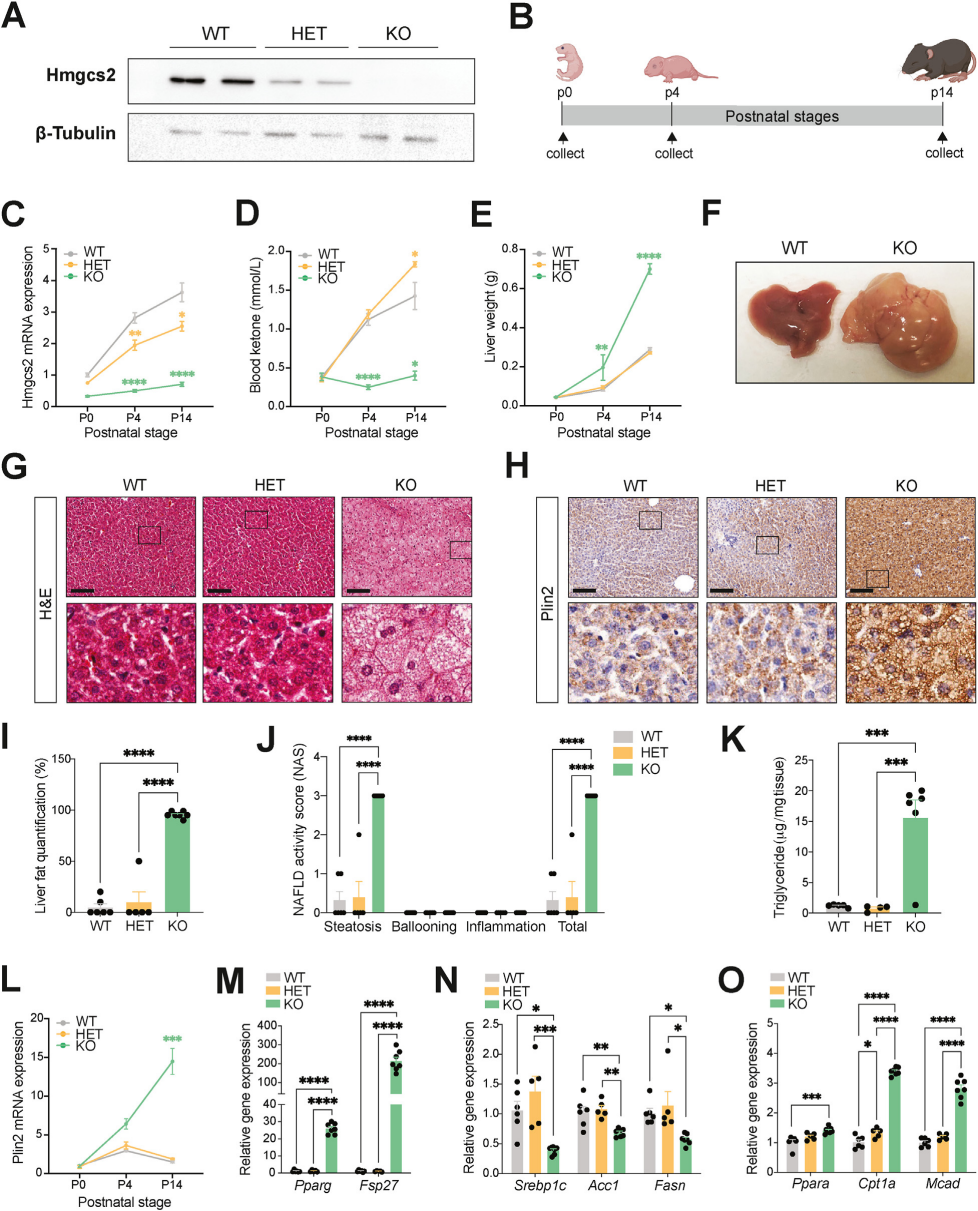
**Ketogenic deficiency through *Hmgcs2* knockout results in fatty liver development in postnatal mice.** (A) Liver Hmgcs2 protein expression in wild-type (WT), *Hmgcs2*-heterozygous (HET) and knockout (KO) mice. (B) Schematic representing the postnatal stages of p0, p4, and p14 at which the mice were examined. (C) *Hmgcs2* gene expression in the liver, (D) blood ketone levels, and (E) liver weights during postnatal development (p0: WT, n = 5; HET, n = 6–9; KO, n = 6; male and female combined/p4: WT, n = 7–9; HET, n = 6–11; KO, n = 4/p14: WT, n = 4–11; HET, n = 3–9; KO, n = 3–10). (F) Representative image of livers of p14 WT and KO mice. (G) H&E and (H) anti-Plin2 IHC stainings of p14 WT, HET and KO mouse liver sections. Scale bar = 100 μm. Boxes indicate regions of higher magnification. (I) Histological liver fat quantification and (J) NAFLD Activity Score (NAS) (WT, n = 6; HET, n = 5; KO, n = 7). (K) Liver triglyceride concentration of p14 WT, HET and KO mice (WT, n = 5; HET, n = 4; KO, n = 6). (L) *Plin2* gene expression in the liver during postnatal development (p0: WT, n = 5; HET, n = 6; KO, n = 6; male and female combined/p4: WT, n = 7; HET, n = 6; KO, n = 4/p14: WT, n = 6; HET, n = 5; KO, n = 7). (M) Lipid accumulation markers (*Pparg*, *Fsp27*), (N) lipid synthesis genes (*Srebp1c*, *Acc1*, *Fasn*) and (O) lipid oxidation genes (*Ppara*, *Cpt1a*, *Mcad*) in p14 WT, HET and KO mouse livers (WT, n = 6; HET, n = 5; KO, n = 7). Data are represented as mean ± SEM. Statistical analysis was performed by one- or two-way ANOVA. ∗*P* ≤ 0.05; ∗∗*P* ≤ 0.01; ∗∗∗*P* ≤ 0.001; ∗∗∗∗*P* ≤ 0.0001. (Created with BioRender.com).

To determine a causal relationship between impaired ketogenesis and NAFLD development *in vivo*, we generated an *Hmgcs2* gene knockout mouse model (*Hmgcs2*-KO) using CRISPR/Cas9-mediated gene targeting (Supplementary Figs. 1A and B). Absence of Hmgcs2 protein expression in the liver confirmed successful knockout of *Hmgcs2* in mice (Figure 2A). Mutant mice exhibited Mendelian frequencies of inheritance, assuming expected ratios to be 25% wild-type (WT), 50% *Hmgcs2* heterozygous (*Hmgcs2*-HET), and 25% *Hmgcs2*-KO from HET × HET crossing (n = 182, χ^2^ = 0.89, P = 0.641) (Supplementary Fig. 1C). Higher lethargy and mortality of *Hmgcs2*-KO mice between postnatal day 14 and 21 (p14 – p21) were observed in preliminary assessments (Supplementary Fig. 1D), supporting the notion that ketone bodies are critical energy substrates during postnatal development, due to the transition from a carbohydrate-to a fat-enriched dietary composition from the prenatal to postnatal period [[13], [14], [15], [16], [17]]. Thus, we examined WT, *Hmgcs2*-HET and *Hmgcs2*-KO mice at three different stages of postnatal development, p0, p4 and p14 (Figure 2B). Gene expression analysis showed that *Hmgcs2* mRNA levels in the liver of WT mice were significantly elevated at p4 and p14, compared to p0 (Figure 2C). Consistently, WT mice exhibited a dramatic increase in blood ketone levels from p0 to p14, representing an increased metabolic demand for hepatic ketogenesis during postnatal development (Figure 2D). However, these elevations in blood ketone levels were not observed in *Hmgcs2*-KO mice, further confirming a functional knockout of ketogenesis. Interestingly, *Hmgcs2*-HET mice exhibited greater (*P* = 0.04) blood ketone levels in comparison to WT mice at p14, despite lower *Hmgcs2* mRNA levels. This might suggest a possible compensatory increase in ketogenesis in the liver. Moreover, no differences in blood glucose levels among WT, *Hmgcs2*-HET and *Hmgcs2*-KO mice were observed (Supplementary Fig. 2A).

Ketogenic deficiency resulted in a mild but significant reduction in growth at p14, as observed by lower body weight of *Hmgcs2*-KO mice (Supplementary Fig. 2B), which appeared to be largely due to a decrease in lean mass (Supplementary Fig. 2C), compared to WT mice. Fat mass, however, was slightly greater in *Hmgcs2*-KO mice compared to both WT (*P* = 0.09) and *Hmgcs2*-HET (*P* < 0.0001) mice. Notably, *Hmgcs2*-KO mouse livers were significantly heavier relative to WT and *Hmgcs2*-HET mice at p4 and p14 (Figure 2E)*.* Also, p14 *Hmgcs2*-KO livers were large, pale, and fattier in comparison to the reddish brown, healthy livers of WT mice (Figure 2F and Supplementary Fig. 2D). H&E and Plin2 staining of liver sections at p14 further confirmed the accumulation of small lipid droplets (microvesicular steatosis) in the hepatocytes of *Hmgcs2*-KO mice (Figure 2G–H). Formal histological evaluation of H&E-stained liver sections revealed higher liver fat quantification and NAFLD activity score (NAS), particularly in the steatosis component, in *Hmgcs2*-KO mice compared to WT and HET mice, while no ballooning and inflammation were observed (Figure 2I–J). Hepatic triglyceride levels were also found to be increased in *Hmgcs2*-KO mice (Figure 2K).

Consistent with the Plin2 immunostaining, *Plin2* mRNA expression was largely increased from p0 to p14 in *Hmgcs2*-KO mice, compared to WT and *Hmgcs2*-HET mice (Figure 2L). Other lipid accumulation gene markers, including *Pparg* and *Fsp27*, were markedly elevated in the livers of *Hmgcs2*-KO mice compared to WT and *Hmgcs2*-HET mice, supporting the extensive fatty liver phenotype in the *Hmgcs2*-KO mice (Figure 2M). On the other hand, *Hmgcs2*-KO mouse livers showed decreased expression of lipid synthesis genes, such as *Srebp1c*, *Acc1*, and *Fasn* (Figure 2N) and increased expression of lipid oxidation genes, including *Ppara*, *Cpt1a*, and *Mcad* (Figure 2O), possibly suggesting a compensatory suppression and activation of these pathways, respectively, to offload the large amounts of lipid accumulation in the liver. Convincingly, this metabolic phenotype in postnatal *Hmgcs2*-KO mice was consistently observed in a very recent study utilizing a different mouse model of *Hmgcs2* gene deletion [45]. Therefore, these results collectively suggest a causal role of impaired ketogenesis, specifically *Hmgcs2* deficiency, in the development of fatty liver disease in postnatal mice, analogous to the rare inborn mitochondrial HMGCS2 (mHS) deficiency disorder, in which infants frequently present with fatty liver [[29], [30], [31], [32]].

### 3.3 A fat-enriched nutritional environment is a prerequisite for fatty liver development in ketogenic deficiency

**Figure 3:**
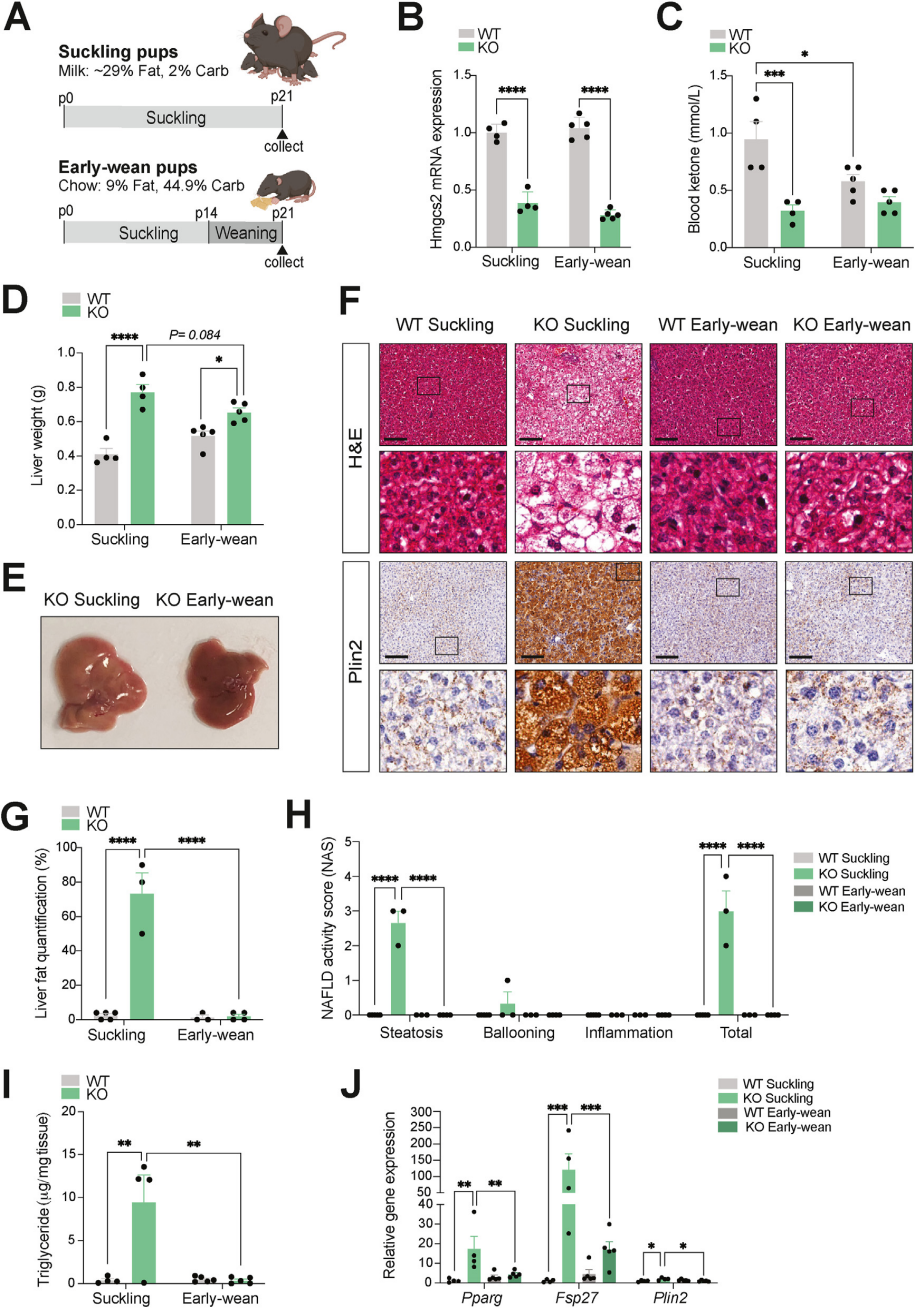
**Fatty liver disease in postnatal *Hmgcs2* knockout mice is rescued by early weaning.** (A) Schematic representation of the early weaning protocol in postnatal WT and *Hmgcs2*-KO mice. Early-wean mice were separated from their mothers at p14 and transitioned from a high-fat breast milk to a standard laboratory chow diet. Suckling control mice remained on breast milk feeding until collection at postnatal day 21 (p21). (B) *Hmgcs2* gene expression in the liver and (C) blood ketone levels of suckling and early-wean WT and *Hmgcs2*-KO mice at p21. (D) Liver weights (suckling WT/KO, n = 4; early-wean WT/KO, n = 5). (E) Representative liver image of *Hmgcs2*-KO suckling and early-wean mice at p21. (F) H&E and anti-Plin2 IHC staining of liver sections. Scale bar = 100 μm. Boxes indicate regions of higher magnification. (G) Histological liver fat quantification and (H) NAFLD activity score (NAS) (suckling WT, n = 5; suckling KO, n = 3; early-wean WT, n = 3; early-wean KO, n = 4; male and female mice combined). (I) Liver triglyceride concentrations of suckling and early-wean WT and *Hmgcs2*-KO mice at p21 (suckling WT/KO, n = 4; early-wean WT/KO, n = 5). (J) Hepatic gene expression analysis of lipid accumulation markers (*Pparg*, *Fsp27*, and *Plin2*). Data are represented as mean ± SEM. Statistical analysis was performed by two-way ANOVA. ∗*P* ≤ 0.05; ∗∗*P* ≤ 0.01; ∗∗∗*P* ≤ 0.001; ∗∗∗∗*P* ≤ 0.0001. (Created with BioRender.com).

Increases in ketone body production and utilization in the postnatal period have been attributed to a surplus of dietary fat provided in the form of breast milk during suckling [[13], [14], [15], [16], [17]]. To determine the contribution of the fat-enriched dietary composition to the development of the fatty liver phenotype in postnatal ketogenic deficient mice, we subjected WT and *Hmgcs2*-KO mice to early weaning (Figure 3A), in order to achieve a transition from the high-fat, low-carbohydrate breast milk (∼29% fat, 2% carbohydrate) to the low-fat, high carbohydrate standard chow diet (9% fat, 44.9% carbohydrate) [16,17,35,36]. Early-wean mice were separated from the mother at p14, a week prior to normal weaning, and were provided with a standard chow diet in the form of soaked cubes, whereas control suckling mice were kept with the mother and continued to have breast milk. The livers of both early-wean and suckling mice were examined at p21. Consistent with the earlier postnatal stages of p0, p4 and p14, hepatic *Hmgcs2* mRNA expression remained significantly low in both suckling and early-wean p21 *Hmgcs2*-KO male mice (Figure 3B). Notably, early weaning decreased (*P* = 0.027) blood ketone levels in WT mice (Figure 3C), suggesting that a change in dietary composition by early weaning reduced the metabolic demand for ketogenesis. As a result, the difference in ketone levels between p21 WT and *Hmgcs2*-KO mice seen at suckling was decreased by early weaning.

Early-wean *Hmgcs2*-KO mice had slightly lower body weights relative to WT mice (Supplementary Fig. 3A), which was consistent with their lean mass, while no difference in fat mass was observed (Supplementary Figs. 3B–C). Early weaning appeared to reduce (*P* = 0.084) the liver weights in *Hmgcs2*-KO mice, which made the weight differences between WT and *Hmgcs2*-KO mouse livers smaller (*P* < 0.001) at early-wean (1.26-fold) than at suckling (1.88-fold) (Figure 3D). Importantly, *Hmgcs2*-KO early-wean mice showed healthier livers in comparison to the enlarged and pale livers of *Hmgcs2*-KO suckling mice (Figure 3E), suggesting reductions in liver fat content. H&E and Plin2 staining of p21 liver sections revealed that, in contrast to the severe microvesicular steatosis seen in *Hmgcs2*-KO mice at suckling, the livers of *Hmgcs2*-KO early-wean mice exhibited a significant reduction in lipid accumulation, making their phenotype comparable to those of WT mice (Figure 3F). Specifically, early weaning significantly removed lipid droplets and likely reduced subsequent lipid accumulation at the center of the liver in *Hmgcs2*-KO mice, while some lipid droplets remained at the periphery of the liver, mainly in the form of macrovesicular steatosis (i.e., large lipid droplets displacing nuclei within hepatocytes) (Supplementary Fig. 3D). Phenotypic reductions in lipid accumulation were consistently observed in early-wean female *Hmgcs2*-KO mice, suggesting no visible sex-specific differences in the role of the postnatal dietary composition and fatty liver development in ketogenesis-deficient mice (Supplementary Fig. 3E). Histological evaluation confirmed reductions in liver fat quantification and NAS steatosis score with early weaning in *Hmgcs2*-KO mice (Figure 3G–H). Liver triglyceride levels of *Hmgcs2*-KO mice were also significantly lowered by early-weaning, making them indistinguishable from those of WT livers (Figure 3I). Consistently, while *Hmgcs2*-KO mice at suckling had greater expression of hepatic lipid accumulation marker genes (*Pparg*, *Fsp27*, *Plin2*) in comparison to WT suckling mice, early weaning significantly lowered this marker gene expression in *Hmgcs2*-KO mouse livers, further supporting an improvement in the fatty liver phenotype (Figure 3J). Together, the phenotypic rescue of hepatosteatosis in postnatal *Hmgcs2*-KO mice by a transition to a low-fat, high-carbohydrate diet suggests that a fat-enriched dietary composition is required for ketogenic deficiency-induced fatty liver disease.

### 3.4 Reduced ketogenesis in adult mice increases susceptibility to NAFLD development

**Figure 4:**
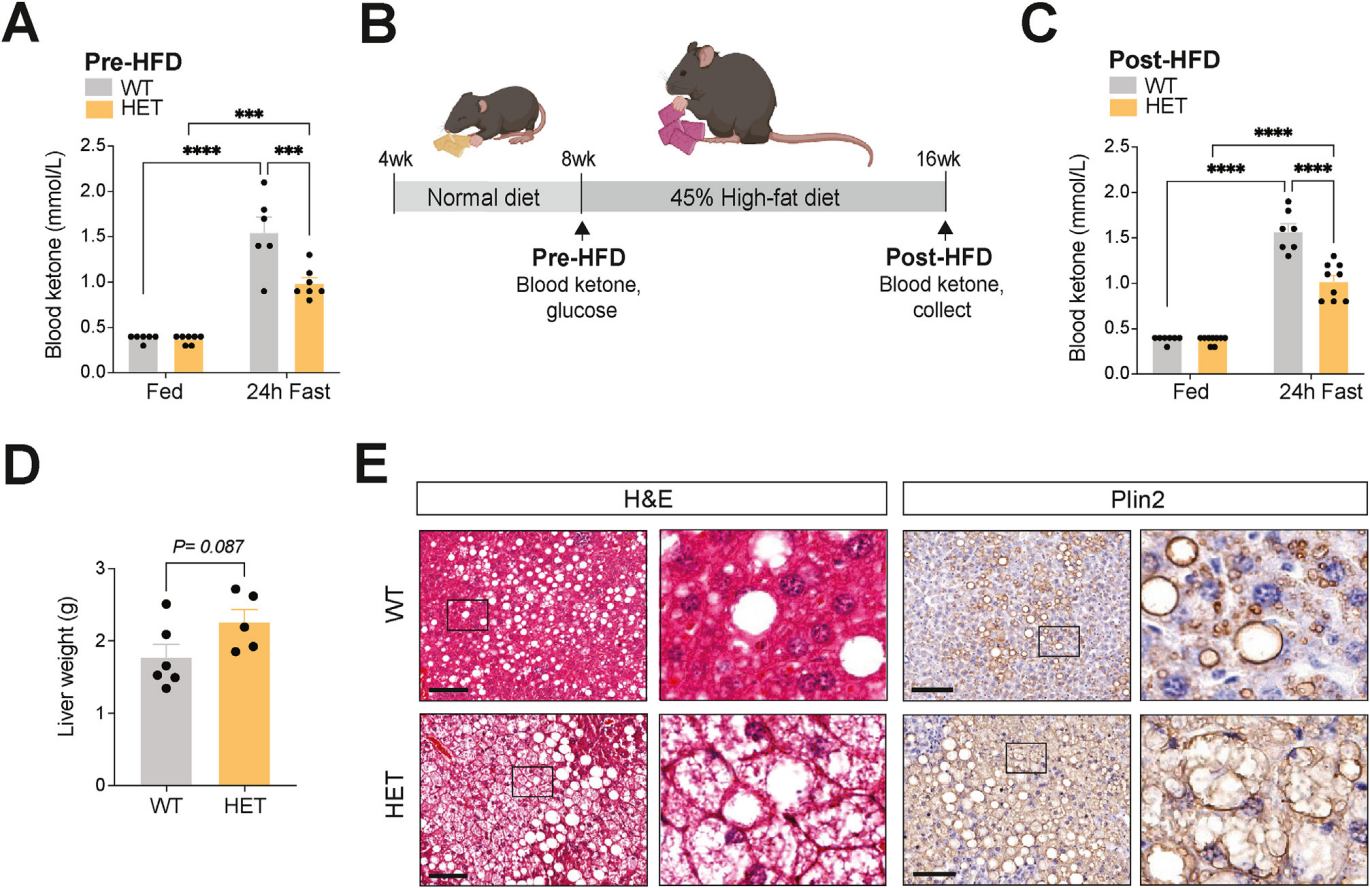
**Ketogenic insufficiency increased the susceptibility of HFD-induced NAFLD development and associated metabolic dysfunction.** (A) Blood ketone levels in 8-week-old fed and 24-hour fasted WT and *Hmgcs2-*HET mice (pre-HFD) (WT, n = 6; HET, n = 7). (B) Schematic for assessment of NAFLD development in WT and *Hmgcs2*-HET mice placed on 8-weeks of HFD. (C) Blood ketone levels in fed and 24-hour fasted post-HFD WT and *Hmgcs2*-HET mice (WT, n = 7; HET, n = 9). (D) Liver weights (WT, n = 6; HET, n = 5). (E) Representative H&E and anti-Plin2 IHC staining of liver sections of WT and *Hmgcs2*-HET mice. Scale bar = 100 μm. Boxes indicate regions of higher magnification. Data are represented as mean ± SEM. Statistical analysis was performed by student's *t*-test or two-way ANOVA. ∗∗∗*P* ≤ 0.001; ∗∗∗∗*P* ≤ 0.0001. (Created with BioRender.com).

As the severity of NAFLD is negatively correlated with ketogenic function [26], and HFD-induced NAFLD mice show insufficient ketogenesis with reduced Hmgcs2 expression, we next questioned whether the degree of ketogenic function and *Hmgcs2* gene dosage affect the progression of NAFLD. Interestingly, while blood ketone levels at weaning (p21) were indistinguishable between WT and *Hmgcs2*-HET mice (Supplementary Fig. 4A), 8-week-old *Hmgcs2*-HET mice had significantly lower fasting-induced blood ketone levels compared to WT mice (Figure 4A), suggesting that reduced *Hmgcs2* gene dosage decreased ketogenic function in adult mice. No differences in fed and fasted glucose levels were observed (Supplementary Fig. 4B). As this phenotype in *Hmgcs2*-HET mice provides a means to test the effect of reduced ketogenesis on NAFLD development, we subjected 8-week-old *Hmgcs2*-HET mice and littermate WT mice to 45% HFD for 8 weeks (Figure 4B). Upon HFD feeding, *Hmgcs2*-HET mice gained slightly more body weight compared to WT mice, which was mainly attributed to increases in fat mass as well as possibly a modest increase in lean mass (Supplementary Figs. 4C–E). A difference in fasting-induced ketogenesis between WT and *Hmgcs2*-HET mice was maintained after HFD feeding (Figure 4C), comparable to the values observed at 8-weeks-old. The livers of *Hmgcs2*-HET mice were slightly heavier than WT mice (*P* = 0.087) (Figure 4D). Importantly, H&E and Plin2 stainings of liver sections showed greater hepatosteatosis, specifically microvesicular steatosis, in *Hmgcs2*-HET mice compared to WT mice (Figure 4E). Together, these findings demonstrate that the degree of ketogenic function, specifically gene dosage of *Hmgcs2*, contributes to the pathogenesis of NAFLD in adult mice.

### 3.5 Increased ketogenesis through *HMGCS2* overexpression improves HFD-induced NAFLD in mice

Next, we tested whether increased ketogenesis through modulation of Hmgcs2 is sufficient to improve the NAFLD condition in mice. A previous study has demonstrated in an *in vitro* model that *HMGCS2* overexpression (OE) in HepG2 hepatocytes can sufficiently activate ketogenesis and increase fatty acid oxidation [46]. Thus, to drive ketogenesis *in vivo*, we intravenously injected HFD-induced NAFLD mice with adenovirus overexpressing human *HMGCS2* (Ad-*HMGCS2*) (Figure 5A), which has a 95% protein sequence homology to mouse *Hmgcs2*. A *GFP*-expressing adenovirus (Ad-*GFP*) was used for control mice. Human *HMGCS2* mRNA expression was predominantly increased in the liver, with slight elevations in heart and brown adipose tissue, in Ad-*HMGCS2* mice compared to Ad-*GFP* mice, suggesting efficient liver-targeting by the adenovirus (Figure 5B, and Supplementary Fig. 5A). In contrast, mouse *Hmgcs2* mRNA expression was reduced in the livers of Ad-*HMGCS2* mice (*P* = 0.007) with no changes in other metabolic tissues (Supplementary Fig. 5B), suggesting a plausible compensatory suppression of endogenous hepatic *Hmgcs2* as a result of human *HMGCS2*-OE. As a result, a mild increase in total HMGCS2 protein level (1.7-fold, *P* = 0.027) was detected through western blot quantification (Supplementary Fig. 5C), possibly owing to the decreased mouse *Hmgcs2* expression and the lack of specificity of the anti-Hmgcs2 antibody in detecting differences between mouse and human isoforms. Importantly, Ad-*HMGCS2* mice exhibited significantly higher blood ketone (β-OHB) levels after 6-hour (1.18 mmol/L) and 24-hour fasting (0.93 mmol/L), whereas Ad-*GFP* mice showed a suppressed ketogenic response (0.65 mmol/L) (Figure 5C). This data suggests a functional elevation of ketogenic activity by *HMGCS2-*OE in HFD-induced NAFLD mice. In addition, a fasting-induced decrease in blood glucose, which was blunted in HFD-fed Ad-*GFP* mice, was improved (*P* < 0.001) in Ad-*HMGCS2* mice at 4 days post-injection, prior to noticeable phenotypic changes (e.g., body weight) (Figure 5D), suggesting the implication of the ketogenic pathway in glucose metabolism, as previously shown [34].

*HMGCS2-*OE in 34-weeks of HFD-induced NAFLD mice led to gradual reductions in body weight at 2- and 3-weeks post-virus injection compared to control (Figure 5E), which was mainly attributed to decreases in fat mass, not lean mass (Figure 5F,G). This body weight loss might possibly be contributed by mild reductions in cumulative food intake at 2- and 3-weeks post-virus injection (*P* = 0.052) in Ad-*HMGCS2* mice, compared to Ad-*GFP* mice (Supplementary Fig. 5D). Remarkably, intraperitoneal GTT and ITT performed both pre- and post-virus injection revealed that glucose handling and insulin sensitivity were improved in Ad-*HMGCS2* mice compared to Ad-*GFP* mice (Figure 5H,I). Significantly lower 6-hour fasting blood glucose, plasma insulin and HOMA-IR in Ad-*HMGCS2* mice further supported improvements in overall glucose homeostasis, while no changes in plasma glucagon levels were observed (Figure 5J–M).

**Figure 6:**
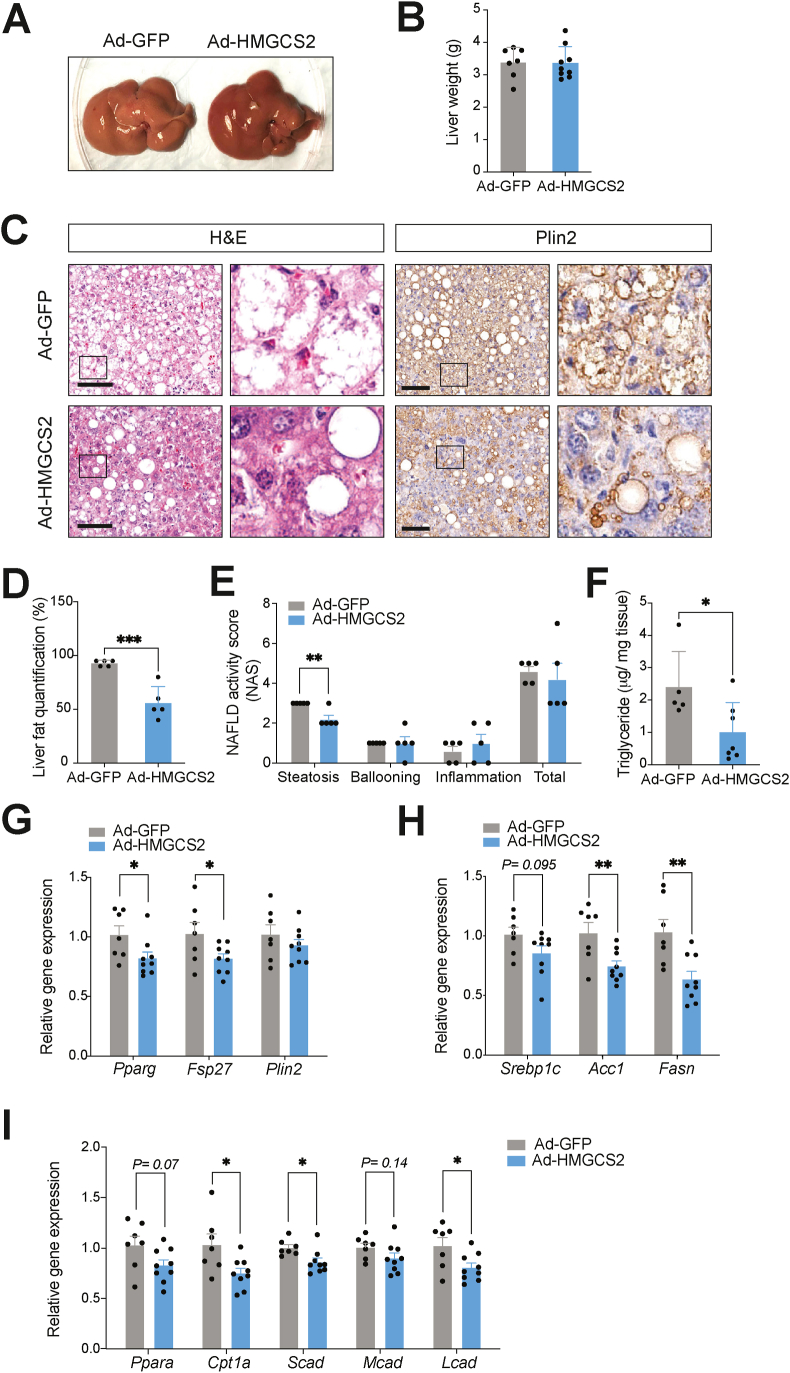
**Ketogenesis activation through *HMGCS2* overexpression improves hepatosteatosis in HFD-induced NAFLD mice.** (A) Representative image of Ad-*GFP* and Ad-*HMGCS2* mouse livers. (B) Liver weights at 3 weeks post-virus injection (Ad-*GFP*, n = 7; Ad-*HMGCS2*, n = 9). (C) H&E and anti-Plin2 IHC staining of liver sections. Scale bar = 100 μm. Boxes indicate regions of higher magnification. (D) Histological liver fat quantification (n = 5/group). (E) NAFLD activity score (NAS) (n = 5/group). (F) Liver triglyceride concentrations of Ad-*GFP* and Ad-*HMGCS2* mice (Ad-*GFP*, n = 5; Ad-*HMGCS2*, n = 7). Gene expression analysis of (G) lipid accumulation (*Pparg*, *Fsp27*, *Plin2*), (H) lipid synthesis (*Srebp1c*, *Acc1*, *Fasn*) and (I) lipid oxidation (*Ppara*, *Cpt1a*, *Scad*, *Mcad*, *Lcad*) (Ad-*GFP*, n = 7; Ad-*HMGCS2*, n = 9) markers. Data are represented as mean ± SEM. Statistical analysis was performed by student's *t*-test. ∗*P* ≤ 0.05; ∗∗*P* ≤ 0.01; ∗∗∗*P* ≤ 0.001.

It was notable that, at collection (3 weeks post-injection), livers of Ad-*HMGCS2* mice looked healthier in contrast to the pale and fatty livers of Ad-*GFP* control mice (Figure 6A), although no difference in liver weight was observed (Figure 6B). H&E and Plin2 staining of liver sections showed an improvement in hepatosteatosis in Ad-*HMGCS2* mice, specifically a marked reduction in the advanced microvesicular steatosis seen in Ad-*GFP* mice (Figure 6C). Formal histological evaluation revealed lower fat quantification and steatosis score in Ad-*HMGCS2* livers, with no changes in the ballooning and inflammation components of NAS (Figure 6D–E). Moreover, hepatic triglyceride level was consistently decreased with *HMGCS2*-OE (Figure 6F). Corroborating the observed reduction in liver fat content, hepatic gene expression analysis revealed significantly lower marker gene expression of lipid accumulation (*Pparg*, *Fsp27*) (Figure 6G) and lipid synthesis (*Acc1*, *Fasn*) in Ad-*HMGCS2* mice (Figure 6H). Interestingly, hepatic lipid oxidation (*Cpt1a*, *Scad*, *Lcad*) gene expression was also significantly lower in Ad-*HMGCS2* mice (Figure 6I). Consistent with the reduction seen in endogenous *Hmgcs2* levels, this result may indicate a compensatory negative-feedback mechanism involving the PPARα signalling pathway and its downstream targets.

As hepatic fibrosis is the major determinant of liver decompensation in NAFLD patients, we tested whether *HMGCS2*-OE could improve liver fibrosis. However, picrosirius red (PSR) staining and formal histological scoring for liver fibrosis (Supplementary Figs. 6A–B), as well as the measurement of liver collagen levels (Supplementary Fig. 6C) indicated no improvement of hepatic fibrosis with *HMGCS2-*OE. In addition, measurement of plasma alanine aminotransferase (ALT) activity, an indicator of liver injury, showed no difference between Ad-*GFP* and Ad-*HMGCS2* mice (Supplementary Fig. 6D). Collectively, these results suggest that increased ketogenesis by *HMGCS2*-OE effectively improves the HFD-induced hepatosteatosis and associated abnormal glucose metabolism in mice, however, may not reverse liver fibrosis.

### 3.6 *HMGCS2* overexpression *in vitro* improves NAFLD-related lipid accumulation in hepatocytes

**Figure 7:**
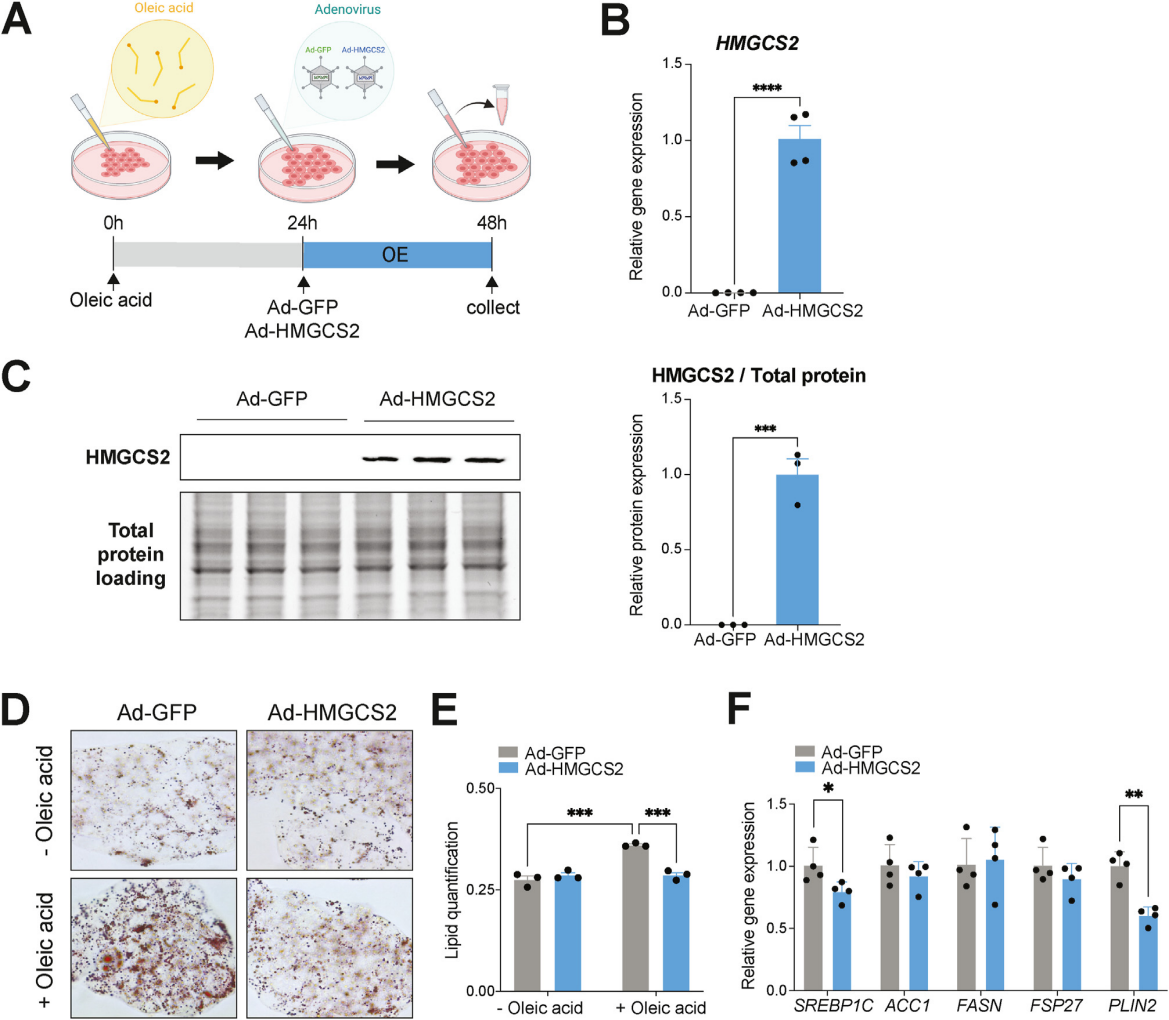
***HMGCS2* overexpression ameliorates lipid accumulation in HepG2 cells.** (A) Schematic representing timeline of *in vitro* experiment, starting with oleic acid treatment at 0-hours, adenovirus-mediated overexpression of *GFP* (Ad-*GFP*) or *HMGCS2* (Ad-*HMGCS2*) at 24-hours and cell collection at 48-hours. (B) RT-qPCR (n = 4/group) and (C) western blot quantification of HMGCS2 expression (n = 3/group) in HepG2 cells after Ad-*HMGCS2* infection, compared to Ad-*GFP*. (D) Representative images of Oil-red-O staining and (E) its quantification of Ad-*GFP* or Ad-*HMGCS2* treated HepG2 cells in the absence (−) and presence (+) of oleic acid (n = 3/group). (F) RT-qPCR of lipid synthesis (*SREBP1C*, *ACC1*, *FASN*) and lipid accumulation (*FSP27*, *PLIN2*) genes in Ad-*GFP* and Ad-*HMGCS2* treated HepG2 cells (n = 4/group). Data are represented as mean ± SEM. Statistical analysis was performed by student's *t*-test or two-way ANOVA. ∗*P* ≤ 0.05; ∗∗*P* ≤ 0.01, ∗∗∗*P* ≤ 0.001; ∗∗∗∗*P* ≤ 0.0001. (Created with BioRender.com).

We next examined the hepatocyte-specific anti-steatotic effect of *HMGCS2*-OE in a cell model of hepatic lipid accumulation. Oleic acid was administered at 0-hours to induce lipid accumulation in HepG2 cells, as done previously [47,48]. 24 hours later, cells were infected with Ad-*GFP* or Ad-*HMGCS2* in the presence of oleic acid, followed by cell collection and assessment after an additional 24 hours (Figure 7A). Elevated HMGCS2 mRNA (*P* < 0.0001) and protein (*P* = 0.0002) expression confirmed successful overexpression (Figure 7B, C). Visualization of neutral lipids with Oil-red O staining and its quantification showed that oleic acid-induced lipid accumulation, seen in Ad-*GFP*-treated control cells (*P* = 0.0001), was markedly reduced with *HMGCS2*-OE (*P* = 0.0002) (Figure 7D–E), validating the comparable reduction of hepatic lipid accumulation seen *in vivo*. Also, consistent with the *in vivo* study, mRNA levels of lipid synthesis (*SREBP1C*) and accumulation (*PLIN2*) marker genes were decreased in *HMGCS2* overexpressed cells (Figure 7F). Together, these findings further support the direct anti-steatotic role of *HMGCS2*-OE in hepatocytes.

## 4 Discussion

In NAFLD, a disruption of energy homeostasis occurs. Perturbations in lipid metabolism, involving excess fatty acid supply and impaired removal, contribute to the buildup of excessive hepatic fat associated with NAFLD [42,43]. Ketogenesis can be activated in response to increased exogenous or endogenous fat in the liver, which can offload significant portions of hepatic fat through the conversion of oxidized fatty acids into energy-carrying ketone bodies for extrahepatic tissues [26,[49], [50], [51], [52]]. However, chronic lipid exposure, such as a prolonged HFD feeding, can lead to ketogenic dysfunction, a plausible mechanism of NAFLD pathogenesis [26,53]. Herein, we demonstrate that NAFLD mice, induced by 32-weeks of HFD feeding, present with significant reductions in fasting-induced blood ketone levels and hepatic Hmgcs2 expression, indicating insufficient activation of the ketogenic pathway. Among all other enzymes in the ketogenic pathway, *Hmgcs2* mRNA and protein levels were significantly increased with fasting, predominantly in the liver, which was dysregulated with HFD-induced NAFLD. Together, these findings support the master regulatory role of Hmgcs2 in hepatic ketogenesis [33]. Importantly, while the impaired ketogenic response of HFD-fed mice shown in our study has been previously replicated [53], other studies have shown an elevation in ketone body levels and hepatic *Hmgcs2* mRNA expression in mice fed 16-weeks of HFD [11,12,53]. This discrepancy can be mainly attributed to the durations of HFD exposure (32 weeks vs. 16 weeks) and the severity in the development of insulin resistance, because, compared to moderate insulin resistance seen in mice fed HFD for 16-weeks [11,12,53], 32-weeks of HFD feeding results in severe insulin resistance in mice [53]. As insulin suppresses Hmgcs2 expression in hepatocytes [54,55], it is reasonable to postulate that higher insulin levels inhibit ketogenesis in the presence of hepatic insulin resistance. This possibility is supported by a recent study, demonstrating that patients with NAFLD and insulin resistance show impaired ketogenesis, which correlates with their degree of intrahepatic lipid accumulation [26]. Diminished ketogenesis has also been previously reported in patients with NASH [56], as well as in obese and diabetic patients [[57], [58], [59]]. Specifically, obese patients with fatty liver have been shown to have reduced total ketone body levels compared to obese patients without fatty liver [28]. These findings suggest that insulin resistance contributes to ketogenic dysfunction in NAFLD, yet HMGCS2 expression has not been causally linked to reduced ketogenesis in patients with NAFLD.

In the present study, our loss- and gain-of-function experiments provide additional evidence to support a causal role of Hmgcs2-mediated ketogenic deficiency and activation in the development and treatment of hepatosteatosis, respectively. In particular, the postnatal fatty liver phenotype seen in our *Hmgcs2*-KO mice was consistently observed in a recent study using a different mouse model of *Hmgcs2* deletion [45], further emphasizing *Hmgcs2* as a critical rate-limiting genetic factor of ketogenesis and hepatic lipid metabolism. This dramatic phenotype is primarily driven by the metabolic demand for hepatic ketogenesis during the postnatal period. From approximately a few hours to 2-weeks post-birth, breast milk is the only source of exogenous energy for rodents. This high-fat to low-carbohydrate dietary ratio of breast milk (fat: ∼29% and carbohydrate: ∼2%) [60] activates hepatic ketogenesis in neonates [13,14,17], which is demonstrated by elevations in blood ketone bodies and hepatic *Hmgcs2* mRNA levels in WT postnatal mice from p0 to p14 seen in our study and from embryonic day 18.5 (E18.5) to p7 as shown in another study [45]. However, in the absence of functional ketogenesis, this postnatal fat-enriched dietary composition leads to the pathogenic accumulation of fat in the liver via insufficient disposal of excess fat-derived acetyl-CoA through the TCA cycle, as demonstrated by our study and another recent study using a different *Hmgcs2*-KO mouse model [45].

We achieved a nearly complete rescue of the severe lipid accumulation in postnatal *Hmgcs2*-KO mouse livers by altering the nutritional environment, suggesting an interrelation between ketogenesis and a fat-enriched dietary composition in the development of the fatty liver. In comparison to the ketogenic composition of breast milk, the standard chow diet is low in fat (∼9%) and high in carbohydrates (∼44.9%). As such, early weaning at p14, one week prior to the normal weaning at ∼ p21 [16], resulted in a substantial decrease in hepatic fat in *Hmgcs2*-KO mice, confirmed by histological, biochemical and molecular examinations of lipid accumulation. In particular, a reduction in Plin2 immunostaining, which emphasizes small lipid droplets [61], depicted a shift from diffuse microvesicular steatosis to focal macrovesicular steatosis at liver tissue periphery in early-wean *Hmgcs2*-KO mice, associated with better clinical outcomes of fatty liver [62].

Our finding described herein of ketogenesis-deficient postnatal *Hmgcs2*-KO mice provide a successful preclinical model of the human condition of mHS deficiency, an inborn error of ketone body metabolism. These genetic errors of ketone metabolism are a subgroup within FAO disorders, and involve deficiencies in enzymes of ketogenesis (i.e., HMGCS2, HMGCL) and ketolysis (i.e., ACAT1, OXCT1/SCOT, MCT1/SLC16A1). Although patients are often asymptomatic, they can rapidly develop severe, life-threatening conditions upon periods of fasting or sickness, generally within their first year of life [[63], [64], [65]]. Hypoketotic hypoglycemia and fatty liver are common clinical presentations of mHS and mitochondrial HMGCL (mHL) deficiencies [29,66]. Treatment and management of these conditions often involve reducing the utilization of FAO and ketogenesis pathways associated with these enzyme deficiencies. For acute treatment, an intravenous glucose infusion is commonly administered to patients, which results in the rapid secretion of insulin that subsequently suppresses adipose lipolysis-mediated fatty acid delivery to the liver for FAO and ketogenesis. For chronic management, patients are often advised to avoid fasting for long periods and reduce their high-fat dietary content [64,67]. Patients with mHL deficiency have also been advised to supplement with carbohydrate-rich diets with moderate protein restriction, specifically leucine, which feeds into the alternative branched-chain amino acid ketogenic pathway and involves HMGCL activity [64,67,68]. Additionally, in one previous study, a patient with carnitine acylcarnitine transferase (CACT) deficiency, a long-chain FAO disorder, was treated with low-fat skim milk, replacing the high-fat breast milk, and showed normal growth and development following one year of treatment without additional metabolic episodes [69]. To our knowledge, a similar reduction in milk fat content has not yet been clinically implemented for inborn errors of ketogenesis, including mHS and mHL deficiencies. Thus, our early-weaning study in postnatal ketogenic deficient mice suggests that a modified form of this treatment may be helpful in treating mHS and mHL patients, especially those presenting with fatty liver. As breast milk contains important nutrients for the development of the newborn [70], complete removal of the breast milk diet may not be feasible, instead defatted breast milk [71] or occasionally supplementing with low-fat milk formulations and high-carbohydrate dietary alternatives to reduce the overall intake of the high-fat breast milk, can be tested. Moreover, supplementation with ketone ester formulations, which have previously shown benefits in patients with FAO disorders (i.e., MAD, CACT, CPT2 deficiencies) [[72], [73], [74]] and inborn errors of ketogenesis (i.e., mHL deficiency) [74], as well as a low-fat maternal diet during pregnancy and lactation, would be interesting avenues to explore in the treatment and prevention of fatty liver in our postnatal ketogenic deficient mice. Overall, these findings highlight the importance of the ketogenic pathway and the macronutrient dietary composition as crucial contributors to the development of fatty liver in postnatal mice.

The implication of ketogenesis in the development and progression of fatty liver disease is further supported by our study using *Hmgcs2*-HET mice that exhibited reduced ketogenic function. Along with their underlying ketogenic insufficiency, exogenous HFD intake and the increased age of the adult mice in comparison to postnatal mice, may all be compounding factors in the development of metabolic dysfunction, as the prevalence and severity of NAFLD and other metabolic conditions (i.e., T2D) increase in adulthood [75,76]. For example, while WT and *Hmgcs2*-HET mice were indistinguishable at the postnatal stage, upon HFD, adult *Hmgcs2*-HET mice developed severe hepatosteatosis compared to WT mice. This indicates that one functional *Hmgcs2* allele is insufficient for proper ketogenesis in the presence of a fat-enriched diet, leading to NAFLD susceptibility. Similarly, adult *Hmgcs2*-ASO mice with ketogenic insufficiency develop a more severe form of the NAFLD-like phenotype with increased hepatic inflammation and fibrosis, seen only upon HFD and not on a chow diet [33]. Thus, our study suggests that reduced ketogenesis may predispose to fatty liver development and metabolic complications in adulthood. As such, the measurement of fasting ketone levels, currently not a standard diagnostic method of screening metabolic disorders, could be useful in identifying patients who are susceptible to fatty liver disease development and associated liver diseases. Moreover, as NAFLD pathogenesis has been associated with genes encoding mediators of lipid metabolism (i.e., *PNPLA3*, *TM6SF2*) [77,78], it would be interesting to examine a genetic link associated with ketogenic mediators (i.e., *HMGCS2* and *HMGCL*).

Our *HMGCS2*-OE study suggests that ketogenic activation may be a possible therapeutic strategy in alleviating hepatosteatosis as an effective lipid disposal pathway. Overexpression of the human homolog of *HMGCS2* in mice resulted in increases in fasting blood ketone levels (6-hour fast, 1.18 mM; 24-hour fast, 0.93 mM) and led to significant reductions in blood glucose and hepatic fat, indicating overall improvements in the fatty liver and metabolic conditions caused by long-term HFD feeding. A decrease in lipid accumulation in *HMGCS2*-overexpressed HepG2 cells further confirmed a hepatocyte-specific ketogenic role in lipid metabolism. Moreover, our findings suggest a possible mechanism underlying the protective effects of dietary interventions against NAFLD, as a ketogenic diet [23] and intermittent fasting [39,79,80] are known to activate ketogenesis. Although no liver-targeting pharmacotherapeutics against NAFLD are presently in use, current drugs, such as metformin [81], GLP1R agonists [3] and SGLT2 inhibitors [4], being clinically used for the treatment of other metabolic diseases (i.e., diabetes), have shown benefits in alleviating different aspects of NAFLD pathology [4,82]. In particular, increased ketogenesis has been suggested as a mechanism behind the benefits of SGLT2 inhibitors [83]. Therefore, it would be informative to test whether the metabolic benefits of dietary interventions and SGLT2 inhibitors against NAFLD are mediated via Hmgcs2 activation.

In addition to improved hepatosteatosis, *HMGCS2*-OE mice exhibited improved glucose homeostasis and reduced body weight with decreased fat mass. A mild decrease in cumulative food intake (∼10%) was observed in *HMGCS2*-OE mice, which is possibly supported by a previous study showing food intake suppression by β-OHB infusion into the hypothalamus [84]. While a pair-feeding study would be required to test whether mildly decreased energy intake could fully account for reduced body weights and improved glucose metabolism seen in *HMGCS2*-OE mice, we found that fasting-induced decreases in blood glucose were restored in *HMGCS2*-OE mice at day 4 post-virus injection, prior to body weight changes (Figure 5D), suggesting that Hmgcs2-mediated ketogenesis regulates glucose metabolism independent of body weight regulation. This finding is aligned with a previous study demonstrating that *Hmgcs2* knockdown led to an increased glycogen pool and augmented glycogen-sourced hepatic glucose production [34]. Future studies using the hyperinsulinemic clamp and isotope tracing metabolomic approaches in *HMGCS2*-OE mice may enhance our understanding of the role of hepatic ketogenesis in glucose homeostasis.

We tested a possibility of whether *HMGCS2* OE-mediated improvement against hepatosteatosis was mediated via the non-ketogenic, ‘moonlighting’ effect of HMGCS2, particularly transcriptional regulation via direct interaction with PPARα, a key transcription factor of FAO, thereby promoting its target gene expression [9,19,[85], [86], [87]]. However, contrary to previous findings [85,86], we found that PPARα downstream target genes involved in FAO (e.g., *Ppara*, *Cpt1a*, *Mcad*) were upregulated in *Hmgcs2*-KO livers (Figure 2O) and downregulated in *HMGCS2*-overexpressed livers (Figure 6I). Notably, elevated PPARα target gene expression has been consistently observed in *Hmgcs2*-ASO-treated mice [33] and other *Hmgcs2* KO mice [45]. While our data and others collectively raise a question about the function of Hmgcs2 as a co-activator of PPARα in the liver, it is possible that modulations of PPARα target genes by *Hmgcs2*-KO or *HMGCS2*-OE might be compensatory responses, regulated by altered metabolites from the dysregulated or active ketogenic pathway.

Liver inflammation and fibrosis were not improved by *HMGCS2*-OE. While we conclude that *HMGCS2*-OE does not have a statistically significant effect on liver fibrosis and inflammation in NAFLD, there was, however a trend towards increased hepatic fibrosis and liver injury markers in Ad-*HMGCS2* mice. Notably, exogenous administration of β-OHB, not AcAc, has been previously described to increase liver fibrosis in mice [88] and inflammatory injury in hepatocytes [89], which may explain the observed trend in the Ad-*HMGCS2* mice. As these results suggest a potentially divergent role of Hmgcs2-mediated ketogenesis in liver fibrosis and inflammation in contrast to lipid and glucose metabolism, careful assessments of the benefits and limitations of ketogenesis-mediated treatments against fatty liver diseases will be necessary. Future studies adapting ketogenic interventions will require considerations for the level and duration of ketogenic activation and resultant concentration of ketone bodies, including the proportion of β-OHB to AcAc.

One limitation of our *HMGCS2*-OE study was the technical difficulty in detecting differences in endogenous mouse Hmgcs2 and ectopic human HMGCS2 protein levels in the liver, due to high sequence conservation (95% homology) and a lack of specific antibodies. Interestingly, despite increased human *HMGCS2* mRNA level and functional evidence of elevated ketogenic activity in *HMGCS2*-OE mice, total HMGCS2 protein expression was only modestly increased. These observations suggest two possibilities. First, as the mRNA level of endogenous mouse *Hmgcs2* was suppressed by exogenous human *HMGCS2*-OE in the liver (Supplementary Fig. 5B), Hmgcs2 expression may be regulated via homeostatic negative feedback upon overexpression, as seen in various regulatory proteins [90]. Second, ectopic human HMGCS2 protein in *HMGCS2*-OE mice may be enzymatically more active, compared to endogenous Hmgcs2 in the control mice. This could be due to differences in post-translational modifications, protein–protein interactions, or enzymatic activities between mouse Hmgcs2 and human HMGCS2. Indeed, it has been shown that HMGCS2 activity is regulated, negatively by acetylation [91] and positively by phosphorylation [92]. HMGCS2 is also found to be highly acetylated in fatty livers [93]. Thus, it is plausible that ectopic HMGCS2 is catalytically more active with less acetylation, whereas long-term HFD feeding acetylates endogenous mouse Hmgcs2, making it less active. Future studies investigating HMGCS2's biology and regulatory mechanism would improve the ketogenesis-based therapeutic strategy against fatty liver disease.

Our data demonstrate the role of Hmgcs2-mediated ketogenesis in liver fat metabolism, yet the liver phenotypes seen in our *in vivo* models could be directly and indirectly influenced by Hmgcs2 in other metabolic tissues. Although the gene expression profiling in key metabolic tissues of mice and humans supports that the liver is the primary ketogenic organ with the highest *Hmgcs2* expression, it is notable that the kidney, small intestine, heart, and adipose tissues showed significantly elevated *Hmgcs2* expression by fasting (Supplementary Figs. 7A and B), consistent with previous observations [[94], [95], [96], [97]]. As such, utilization of tissue-specific *Hmgcs2* knockout and overexpression *in vivo* models will be required in the future to examine the functional implication of extrahepatic ketogenic effects in the development and improvement of fatty liver, respectively. Specifically, kidney- or gut-specific *Hmgcs2*-KO mouse models could provide meaningful information on the role of extrahepatic ketogenesis in the setting of postnatal development and dietary changes in early weaning.

In conclusion, our study demonstrates that Hmgcs2-mediated ketogenesis is a key contributor to NAFLD pathogenesis and treatment. We show that (1) impaired ketogenesis in HFD-induced NAFLD mice is mediated by reduced hepatic expression of Hmgcs2; (2) Hmgcs2-associated ketogenic deficiency and a fat-enriched dietary composition are both required for postnatal fatty liver development; (3) insufficient level of ketogenic function or *Hmgcs2* gene dosage promote adult-onset NAFLD upon HFD; and (4) *HMGCS2* OE-mediated ketogenesis activation improves HFD-induced NAFLD and metabolic dysfunction. Together, our findings suggest impaired ketogenesis and dysregulated hepatic Hmgcs2 expression as possible markers of NAFLD progression and susceptibility, highlighting a possible role of controlled ketogenic activation as a potential therapy for hepatosteatosis.

## Author contributions

Conceptualization, S.A., R.Y.K., and K.-H.K; Methodology, S.A., R.Y.K., and K.-H.K. Investigation, S.A., R.Y.K., T.F., J.S., X.Z., Y.O., A.D., A.C., M.D., E.E.M. and K.-H.K.; Resources, K.-H.K.; Writing - Original draft, S.A., and K.-H.K.; Writing – Review & Editing, all authors; Visualization, S.A., and K.-H.K.; Supervision, K.-H.K.; Funding Acquisition, K.-H.K.

## Acknowledgements

This study was supported by the start-up fund from the University of Ottawa Heart Institute to Dr. Kyoung-Han Kim. Dr. Kyoung-Han Kim was also supported by a National New Investigator Award from the Heart and Stroke Foundation of Canada (HSFC). Shaza Asif was supported by the Canada Graduate Scholarships Master Award (CGS-M) by the Canadian Institutes of Health Research, and the Queen Elizabeth II Graduate Scholarship in Science and Technology. Dr. Ri Youn Kim was supported by fellowships from the University of Ottawa Cardiology Research Endowment Fund and the National Research Foundation of Korea. Yena Oh was supported by the Frederick Banting and Charles Best Canada Graduate Scholarships Doctoral Award (CGS-D). Dr. Michael Downey was supported by Natural Sciences and Engineering Research Council of Canada (NSERC) Discovery Grant (RGPIN-2021-03887). The authors are grateful to Dr. Lauryl Nutter and The Centre for Phenogenomics (TCP) for *Hmgcs2* knockout mouse generation. The authors also thank Dr. Hoon-Ki Sung and Dr. Joe Eun Son at the Hospital for Sick Children for their careful and critical reading of the manuscript.

## Conflict of interest

None declared.
