# Supplementary material for: Hmgcs2-mediated ketogenesis modulates high-fat diet-induced hepatosteatosis
Source: Mol Metab. 2022 Apr 12;61:101494. doi: 10.1016/j.molmet.2022.101494 (PMC9039870; doi:10.1016/j.molmet.2022.101494)
Supplement: Multimedia component 1 [file mmc1.docx]

**Supplementary Materials**

**Hmgcs2-mediated ketogenesis modulates high-fat diet-induced hepatosteatosis**

Shaza Asif, Ri Youn Kim, Thet Fatica, Jordan Sim, Xiaoling Zhao, Yena Oh, Alix Denoncourt, Angela Cheung, Michael Downey, Erin E. Mulvihill, Kyoung-Han Kim

**Supplementary Table 1: qPCR primer sequences for gene expression analysis**

| **Gene** | **Forward (5’ – 3’)** | **Reverse (5’ – 3’)** |
| --- | --- | --- |
| Mouse | | |
| *Acat1* | GTCTGGCTAGTATTTGCAACG | TTCAGCCGGTCACATGG |
| *Hmgcl* | GGACTTCATCTGTCAAGCC | TCATTGTATACACCCAATTCCC |
| *Hmgcs2* | GGTGTCCCGTCTAATGGAGA | ACACCCAGGATTCACAGAGG |
| *Bdh1* | GAATTCAGCCTGCCGGTTTG | TGCATCCCGCTGTCAGGTAA |
| *Pparg2* | CACCAGTGTGAATTACAGCAAATC | AGCTGATTCCGAAGTTGGTG |
| *Fsp27* | CTGGAGGAAGATGGCACAAT | GGGCCACATCGATCTTCTTA |
| *Plin2* | GACCTTGTGTCCTCCGCTTAT | CAACCGCAATTTGTGGCTC |
| *Srebp1c* | CGCTACCGGTCTTCTATCAATG | TTGCTTTTGTGTGCACTTCG |
| *Acc1* | ATTGACCCAGACTGGCTTGAA | GTGTGAAGGCTGCTTTGTGAAC |
| *Fasn* | CCCTTGATGAAGAGGGATCA | ACTCCACAGGTGGGAACAAG |
| *Ppara* | CCGCAATGGACCATGTAAC | CAGCTCTAGCATGGCCTTTT |
| *Cpt1a* | GAGACTTCCAACGCATGACA | ATGGGTTGGGGTGATGTAGA |
| *Scad* | ACCAAAGCTTGGATCACCAACTCC | AACCAGGAAGGCACTGATACCCTT |
| *Mcad* | GCTCGTGAGCACATTGAAAA | CATTGTCCAAAAGCCAAACC |
| *Lcad* | TACGGCACAAAAGAACAGATCG | CAGGCTCTGTCATGGCTATGG |
| Human | | |
| *HMGCS2* | GGTGCCTTCTCTTATGGCTC | GACACACACTTTCGGGAGG |
| *FSP27* | GGGATACAGTGTTCATGGTCCT | TCAATCTTCTTGGCAGGCTTATG |
| *PLIN2* | TTGCAGTTGCCAATACCTATGC | CCAGTCACAGTAGTCGTCACA |
| *SREBP1C* | ACAGTGACTTCCCTGGCCTAT | GCATGGACGGGTACATCTTCAA |
| *ACC1* | GCTCCTTGTCACCTGCTTCT | CAAGGCCAAGCCATCCTGTA |
| *FASN* | AGCGGCTCTGAGACCTCGGA | GCAGGCTGTGTCCAGTGCGA |

**
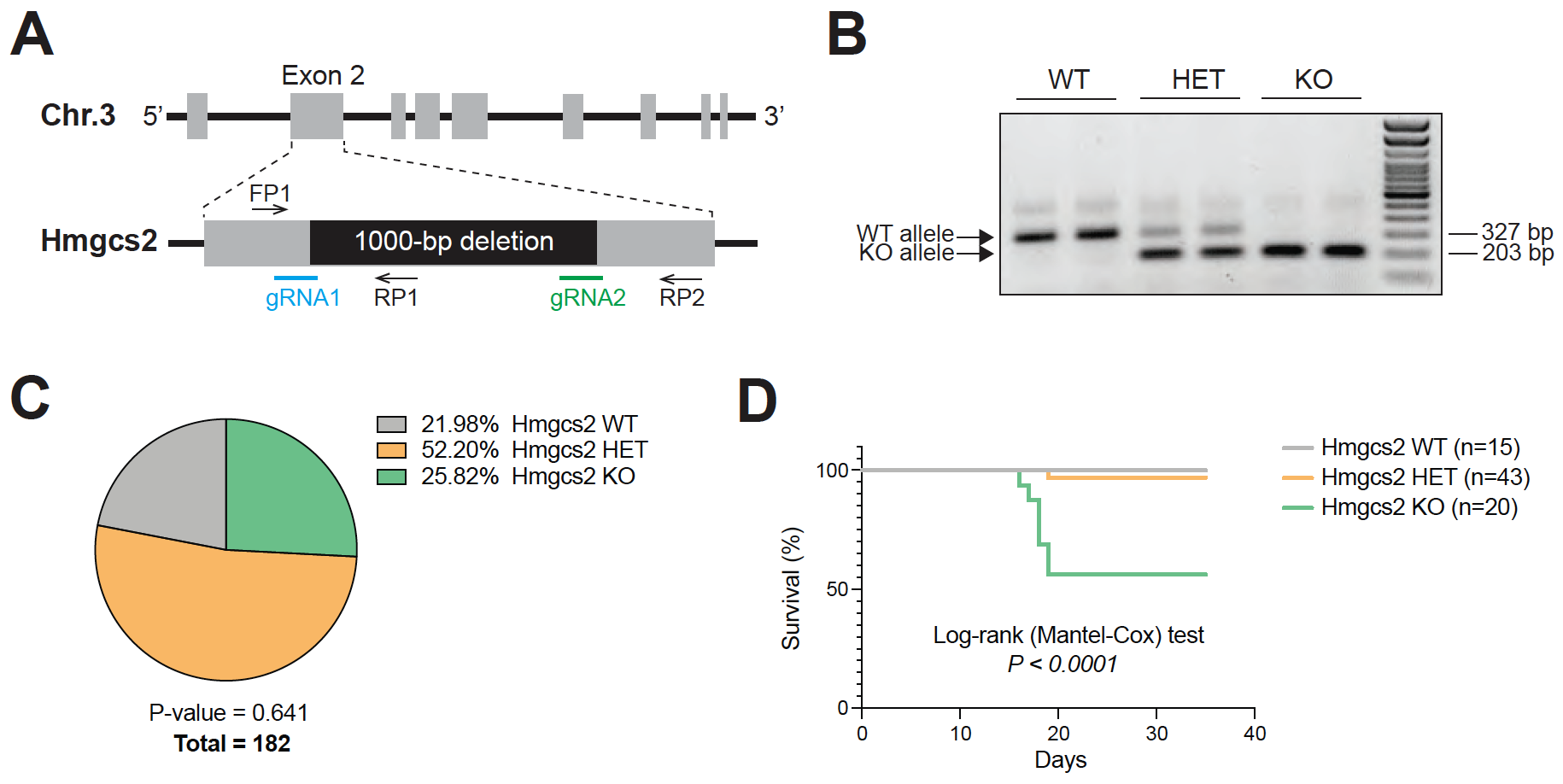
**

**Supplementary Figure 1: *Hmgcs2* knockout mouse generation.** (**A**) CRISPR-Cas9 mediated 1000-bp deletion within exon 2 of *Hmgcs2* on chromosome 3, utilizing a pair of guide RNAs (gRNA1, gRNA2). (**B**) Gel electrophoresis showing wild-type (WT; 327 base-pair) and knockout (KO; 203 base-pair) alleles generated through a polymerase chain reaction using a common forward primer (FP1) and two reverse primers (RP1, RP2) spanning the deletion region. (**C**) Chi-square analysis of Mendelian frequencies of inheritance of *Hmgcs2* mutant mice, assuming expected rations of 25% WT, 50% HET, and 25% KO (n = 182). (**D**) Kaplan-Meier survival analysis of *Hmgcs2* mutant mice with Log-rank (Mantel-Cox) test (WT, n = 15; HET, n = 43; KO, n = 20).


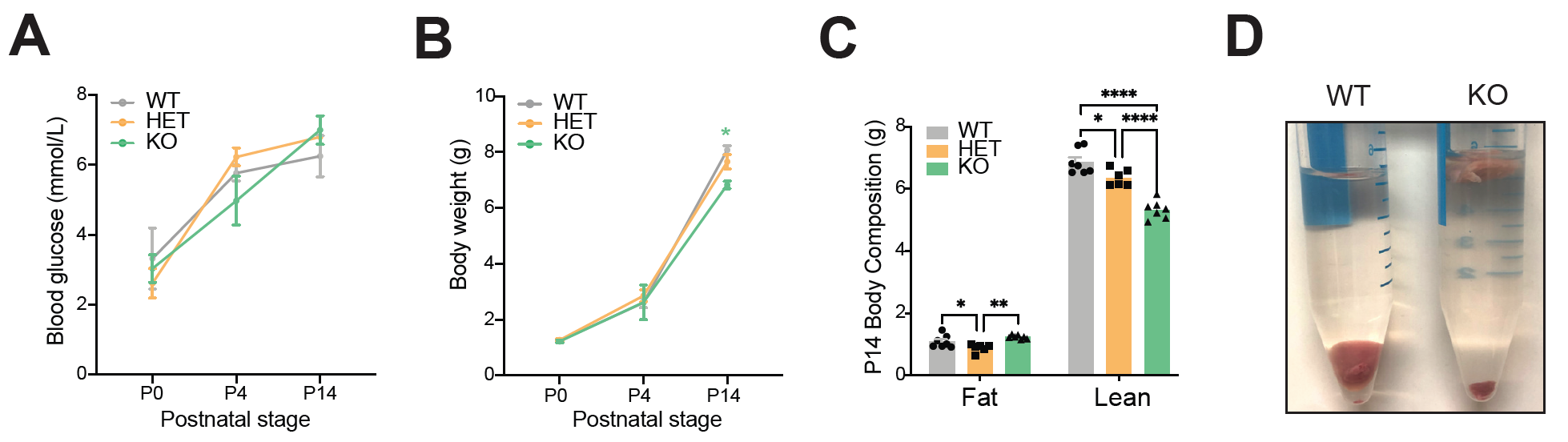


**Supplementary Figure 2: Postnatal *Hmgcs2* knockout mice.** (**A**) Blood glucose levels and (**B**) body weights in WT, HET and KO mice during postnatal development (p0: WT, n = 5; HET, n = 9; KO, n = 6; male and female combined / p4: WT, n = 9; HET, n = 11; KO, n = 4 / p14: WT, n = 4 - 11; HET, n = 3 - 9; KO, n = 3 - 10). (**C**) Body composition analysis showing fat and lean mass at p14 (WT, n= 6; HET, n= 5; KO, n= 6). (**D**) P14 WT and KO mouse livers fixed in 4% paraformaldehyde solution upon collection. Data are represented as mean ± SEM. Statistical analysis was performed by one- or two-way ANOVA. **P* ≤ 0.05; ***P* ≤ 0.01, *****P* ≤ 0.0001.


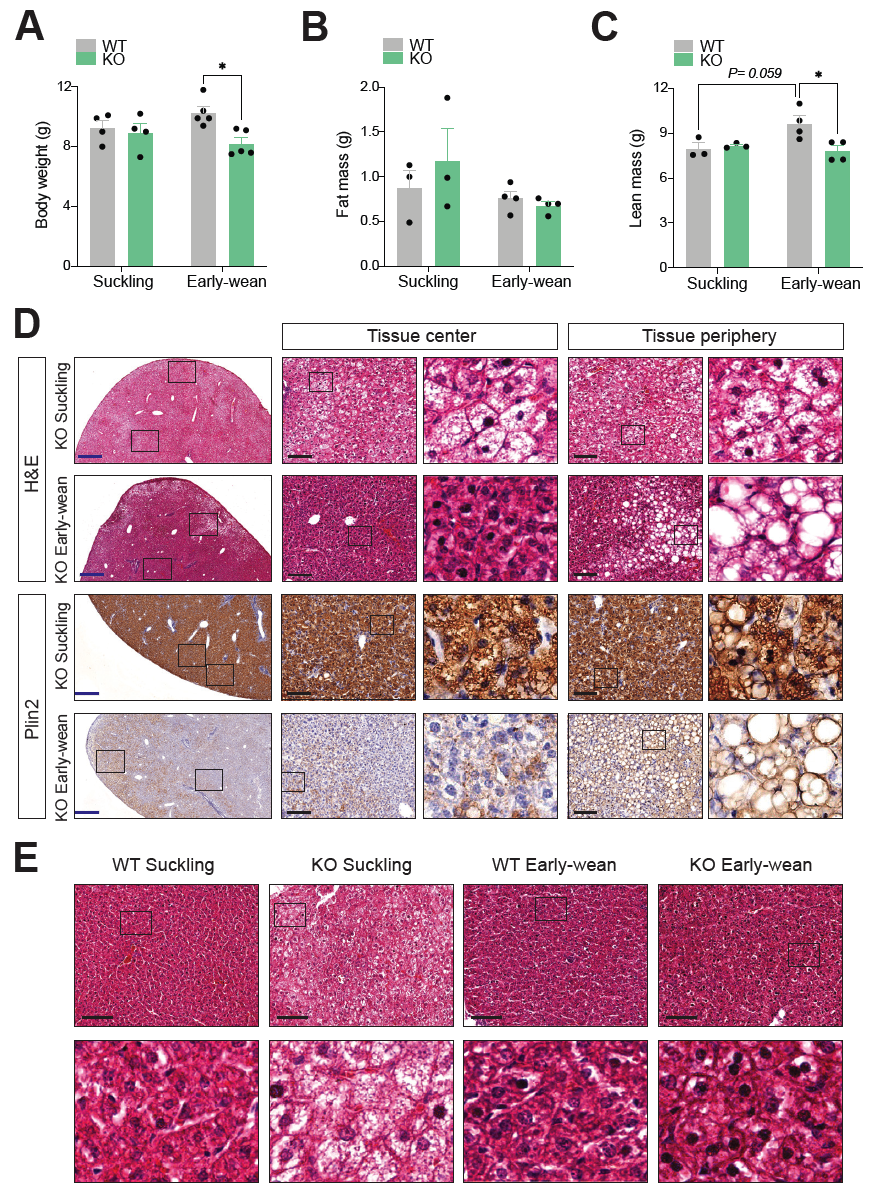


**Supplementary Figure 3: Postnatal *Hmgcs2* knockout early-wean mice.** (**A**) Body weights (suckling WT/KO, n = 4; early-wean WT/KO, n = 5), (**B**) fat mass and (**C**) lean mass of suckling and early-wean WT and *Hmgcs2*-KO mice at p21 (suckling WT/KO, n = 3; early-wean WT/KO, n = 4). (**D**) H&E and anti-Plin2 IHC staining of liver sections of p21 *Hmgcs2*-KO male mice at suckling and early weaning, with magnifications at tissue center and periphery. (**E**) H&E staining of liver sections of p21 WT and *Hmgcs2-*KO female mice at suckling and early-wean. Blue scale bar = 500 μm. Black scale bar = 100 μm. Boxes indicate regions of higher magnification. Data are represented as mean ± SEM. Statistical analysis was performed by the two-way ANOVA. **P* ≤ 0.05.

**
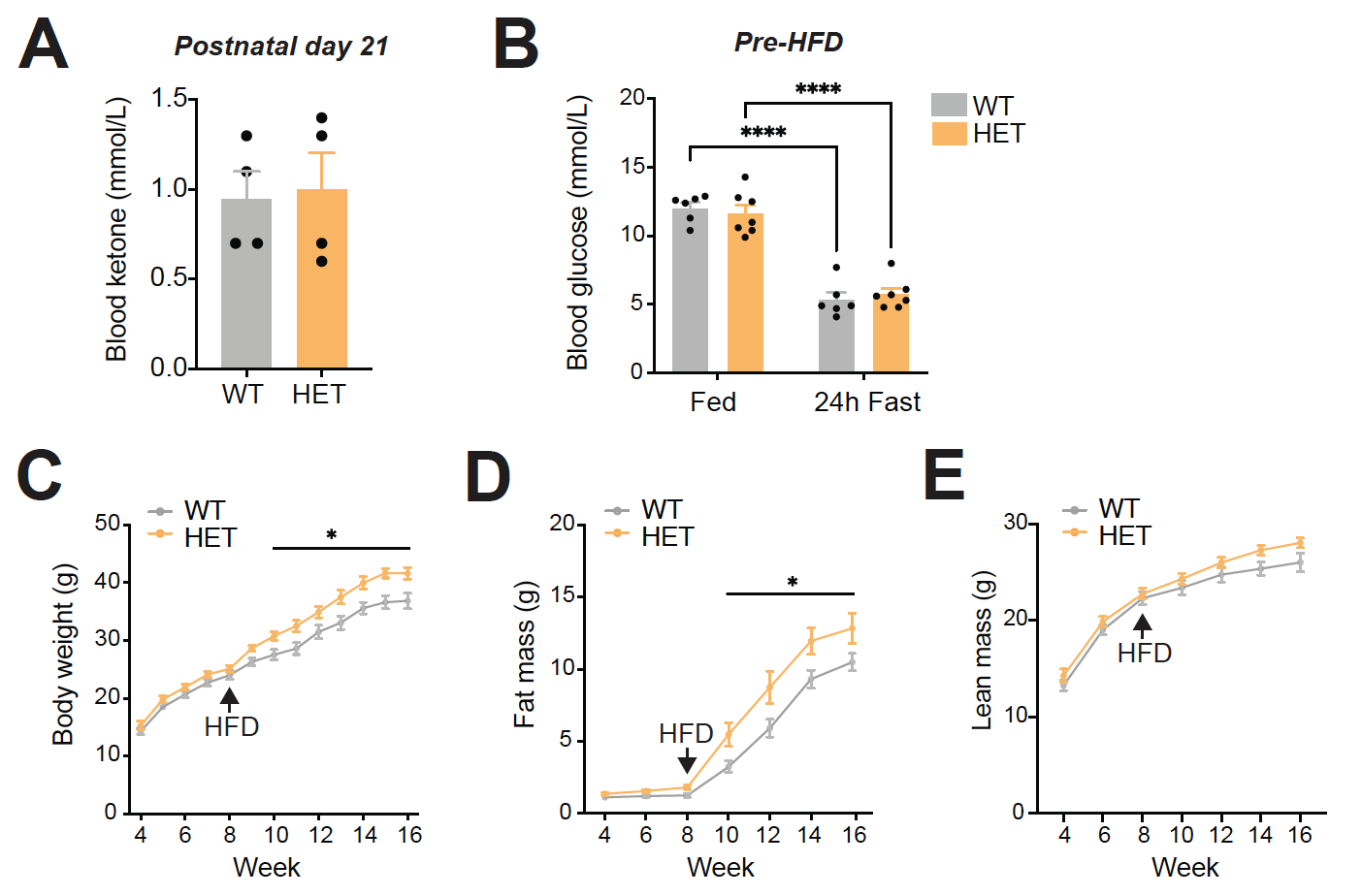
**

**Supplementary Figure 4: Ketogenic insufficient *Hmgcs2*-heterozygous adult mice.** (**A**) Blood ketone levels of p21 WT and *Hmgcs2-*HET male mice (WT, n = 4; HET, n = 4). (**A**) Blood glucose levels in 8-week-old, fed and 24-hour fasted WT and *Hmgcs2-*HET male mice (pre-HFD) (WT, n = 6; HET, n = 7). (**B**) Weekly measurements of body weights (WT, n = 9; HET, n = 9 - 12). Biweekly measurements of (**C**) fat and (**D**) lean mass. Data are represented as mean ± SEM. Statistical analysis was performed by student’s *t*-test two-way ANOVA. **P* ≤ 0.05; *****P* ≤ 0.0001.

**
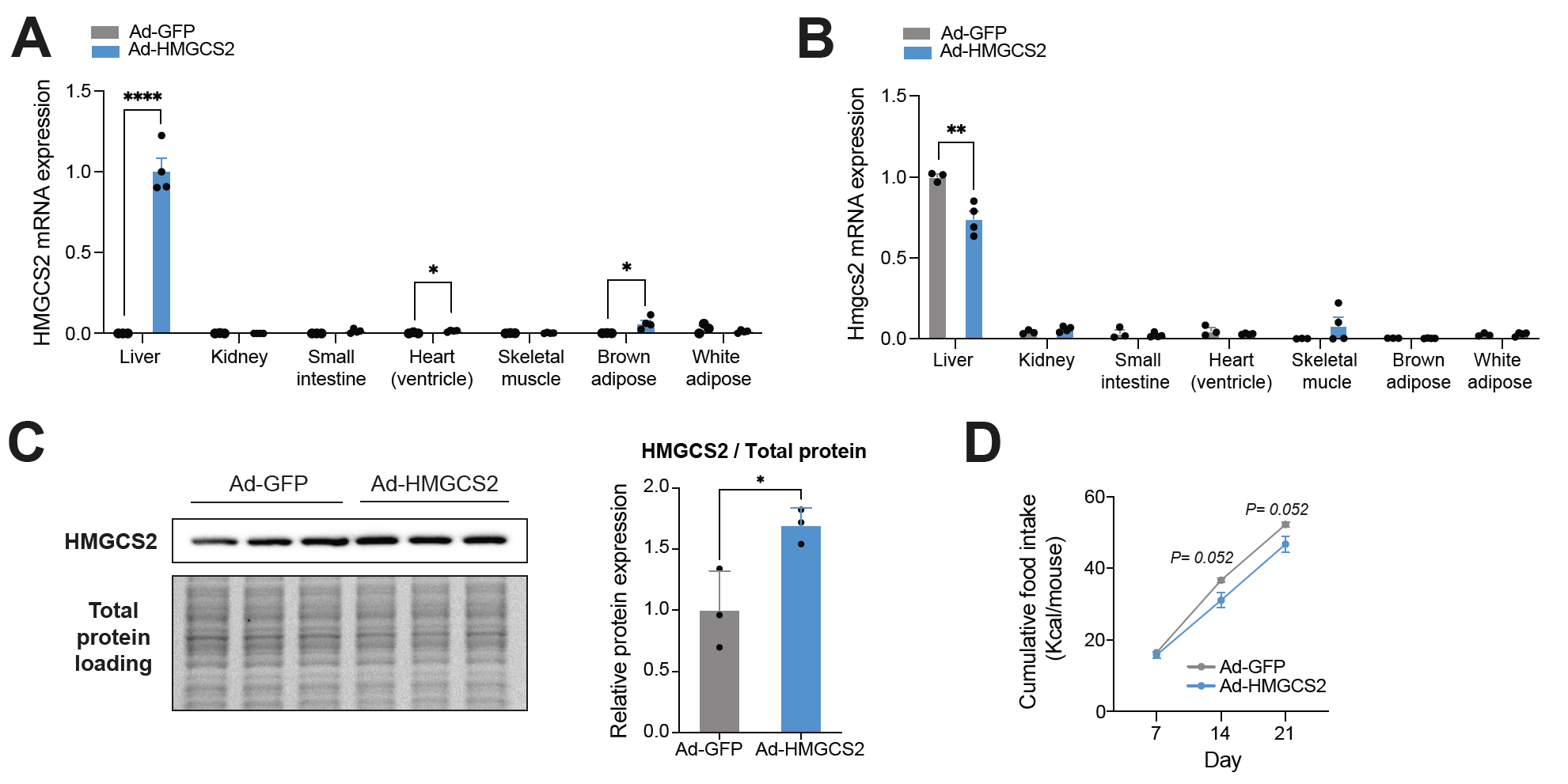
**

**Supplementary Figure 5: Hmgcs2 tissue-gene expression analysis and energy intake of *HMGCS2* overexpression mice.** (**A**) Human *HMGCS2* and (**B**) mouse *Hmgcs2* mRNA expression in metabolic tissues (liver, kidney, small intestine, heart-ventricle, skeletal muscle, brown and white adipose) collected from overnight-fasted (15-hour) Ad-*GFP* and Ad-*HMGCS2* mice (Ad-*GFP*, n = 3; Ad-*HMGCS2*, n = 4). (**C**) Western blot quantification of HMGCS2 protein in the liver tissues collected at 4 days post-virus injection (n = 3/group). (**D**) Cumulative food intake measurement of Ad-*GFP* and Ad-*HMGCS2* mice for 3-weeks post-virus administration (Ad-*GFP*, n = 7; Ad-*HMGCS2*, n = 9). Data are represented as mean ± SEM. Statistical analysis was performed by student’s *t*-test or two-way repeated measures ANOVA. **P* ≤ 0.05; ***P* ≤ 0.01, *****P* ≤ 0.0001.


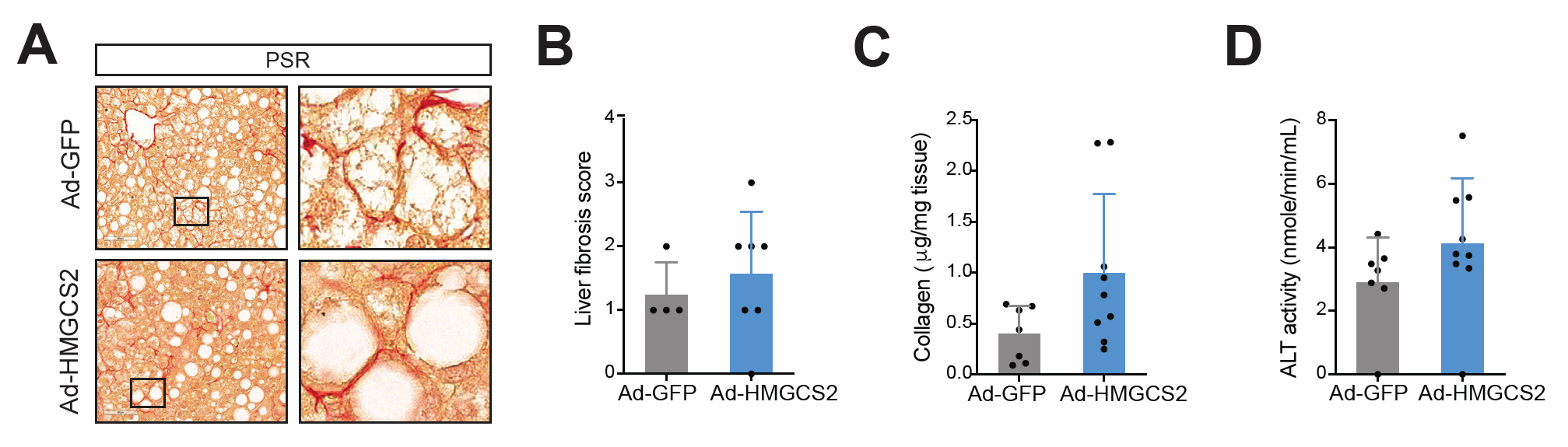


**Supplementary Figure 6:** **Ketogenesis activation through *HMGCS2* overexpression results in no change in markers of hepatic inflammation and fibrosis in HFD-induced NAFLD mice. (A)** Picrosirius red (PSR) staining for fibrosis in Ad-*GFP* and Ad-*HMGCS2* mouse liver sections. Scale bar = 100 μm. Boxes indicate regions of higher magnification. (**B**) Histological liver fibrosis score (Ad-*GFP*, n = 4; Ad-*HMGCS2*, n = 7), (**C**) liver collagen concentrations and (**D**) plasma alanine aminotransferase (ALT) activities in Ad-*GFP* and Ad-*HMGCS2* mice (Ad-*GFP*, n = 7; Ad-*HMGCS2*, n = 9). Data are represented as mean ± SEM. Statistical analysis was performed by student’s *t*-test.


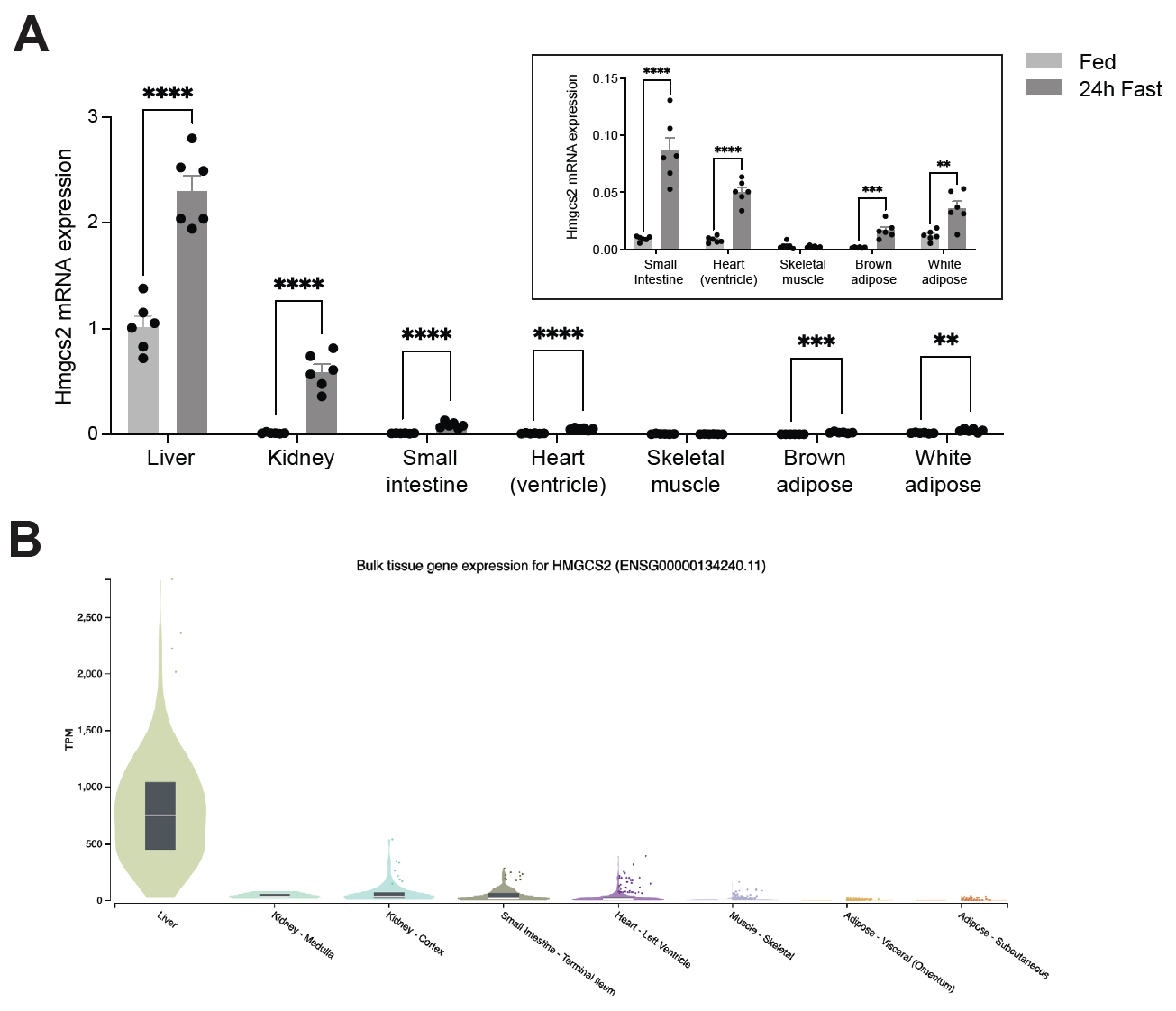


**Supplementary Figure 7: Hmgcs2 gene expression analysis in metabolic tissues**. (**A**) *Hmgcs2* mRNA expression in metabolic tissues (liver, kidney, small intestine, heart-ventricle, skeletal muscle, brown and white adipose) collected from fed and 24-hour fasted 8-week-old C57B6/J mice on standard chow (*n =* 6/group). Inset shows the magnified bar graphs of small intestine, heart-ventricle, skeletal muscle, brown and white adipose. (**B**) RNA-sequencing based gene expression profiling of human *HMGCS2* in metabolic tissues. Data was obtained from the Genotype-Tissue Expression (GTEx) Analysis Release V8 (dbGaP Study Accession: phs000424.v8.p2) (liver, n = 226; kidney-medulla, n = 4; kidney-cortex, n = 85; small intestine-terminal ileum, n = 187; heart-left ventricle, n = 432; muscle-skeletal, n = 803; adipose-visceral, n = 541; adipose-subcutaneous, n = 663). TPM; transcripts per million. Data are represented as mean ± SEM. Statistical analysis was performed by student’s *t*-test. ***P* ≤ 0.01; ****P* ≤ 0.001; *****P* ≤ 0.0001.
